# The relationship of immune cells with autism spectrum disorder: a bidirectional Mendelian randomization study

**DOI:** 10.1186/s12888-024-05927-5

**Published:** 2024-06-27

**Authors:** Congcong Fang, Yonghao Sun, Cuifang Fan, Di Lei

**Affiliations:** 1 Outpatient Department, Renmin Hospital of Wuhan University, Wuhan, 430000 China; 2 Fujian Provincial Key Laboratory of Reproductive Health Research, School of Medicine, Xiamen University, Xiamen, China; 3 Department of Obstetrics, Renmin Hospital of Wuhan University, Wuhan, 430000 China

**Keywords:** ASD, Immunity, Mendelian Randomization, Genetics, SNPs

## Abstract

**Background:**

Observational studies have indicated a correlation between immunological inflammation and the risk of autism spectrum disorder (ASD). However, the causal relationship between immunological inflammation and ASD remains uncertain.

**Methods:**

Immunity-wide data sources were retrieved from the GWAS catalog. Genetic summary data on ASD were retrieved from two independent GWAS. We performed two independent bi-directional, two-sample Mendelian randomization (MR) analyses and a meta-analysis based on the two independent MR estimates to assess the causal relationship between ASD and immune cell signatures.

**Results:**

We have discovered 26 potential correlations between genetic predisposition in the immunophenotypes and ASD. The meta-analysis of the two inverse variance weighted (IVW)-produced estimates provided further evidence supporting the potential causal relationship between immunophenotypes and ASD. Based on the findings of the reverse MR analysis, it was determined that there are two potential negative causal relationships between ASD and immunophenotypes. However, the meta-analysis of the two IVW-derived MR estimates indicated that immunophenotypes were not significantly influenced by ASD (OR = 0.87, 95% CI = 0.73 -1.03, *P* = 0.09; OR = 0.91, 95% CI = 0.81–1.01, *P* = 0.08).

**Conclusions:**

This study expanded immune cell subtypes that were potentially causally associated with ASD risk as well as identified ASD-specific immune cell subtypes. The discovery has the potential to lead to earlier detection and more effective treatment techniques.

**Supplementary Information:**

The online version contains supplementary material available at 10.1186/s12888-024-05927-5.

## Introductions

Autism Spectrum Disorder (ASD) is an intricate and widespread neurodevelopmental disease with an unclear cause. ASD is classified based on behavior and is characterized by impairments in social communication and interaction, as well as the existence of limited and repetitive patterns of behavior, interests, or hobbies [1]. The incidence of ASD has significantly increased in recent decades, affecting 1 in 44 children. Moreover, the prevalence of ASD is more than four times higher in males compared to females [2]. The issue is a significant societal concern and a growing worldwide responsibility that has consequences for public health services [3]. Multiple endeavors have been made to ascertain the cause of ASD, yet it still remains mostly unknown. The development of ASD is believed to be influenced by genetic, neurological, immunological, and environmental factors. Neuroimmunology is receiving increasing focus due to the potential connection between immune response dysregulation and abnormalities in neurodevelopment. Numerous studies have documented altered immune system function in people with ASD [4].

The long-held belief that the brain is an immune-privileged organ has shifted paradigms. In recent years, the interaction between the immune and nervous systems has been well described in a variety of pathological cases, ranging from autoimmune diseases like multiple sclerosis to more traditional cognitive dysfunctions like Alzheimer’s disease [5]. Numerous studies have shown that people with ASD have a different immunological response. Evidence of changes in the functioning of the central and peripheral immune systems suggests that a subgroup of people with ASD exhibit immunological dysregulation [6]. Furthermore, children with ASD have a higher frequency of immune-related comorbidities such as autoimmune disorders, allergies, and psoriasis when compared to healthy controls [7]. Immune system changes include inappropriate immune cell activation, the production of autoantibodies, cytokine and chemokine imbalances, and increased permeability of the blood–brain barrier [8]. Individuals with ASD often exhibit lymphocyte immunomodulation (T lymphocytes, B lymphocytes, natural killer (NK) cells, and dendritic cells). The onset of maternal autoimmune illnesses during pregnancy might significantly influence the likelihood of ASD in the child as well [9]. On the other hand, the current research has shown contradictory conclusions regarding the connection between immunological inflammation and ASD [10]. This might be attributed to factors such as small sample numbers, errors in study design, and the presence of confounding variables that were not well addressed in these investigations. ASD has a high level of complexity and heterogeneity. The topic of whether immunological dysregulation is a fundamental etiology or a later outcome remains unresolved [8].

Mendelian randomization (MR) is a method that mimics a randomized controlled trial and utilizes genetic information as an instrumental variable to assess exposure. Because allele allocation occurs during meiosis and is not influenced by environmental exposures, MR analysis is considered to be less affected by underlying confounding factors and may be used to infer causal relationships, provided the statistical assumptions are satisfied [11]. However, to our knowledge, no MR study has been conducted to evaluate the correlation between immune cells and ASD. To further investigate the causal roles played by peripheral immunity in ASD risk, the present research used two-sample Mendelian randomization (MR) studies to evaluate the possible causal association between immune cells and ASD.

## Methods

### Study design

**Fig. 1:**
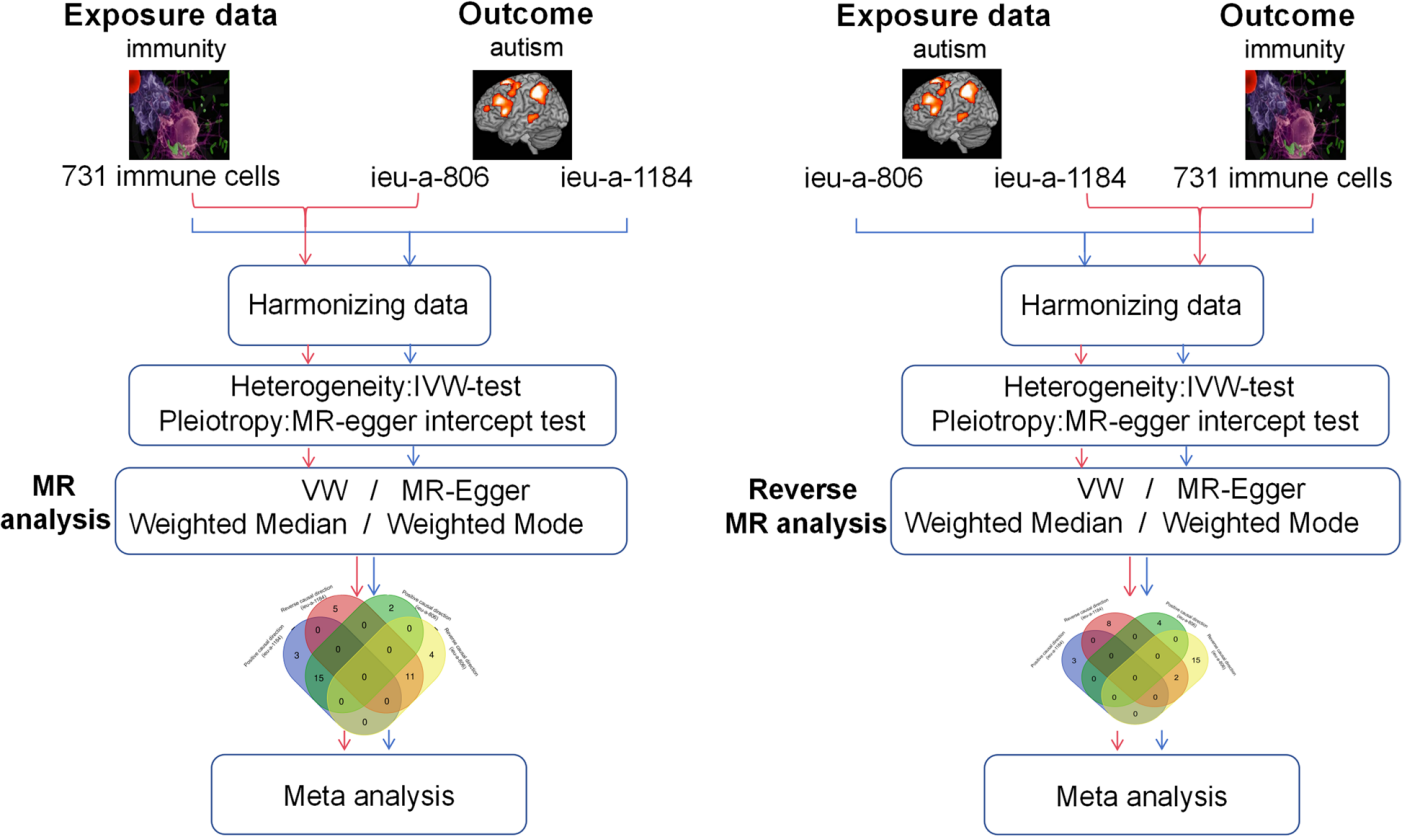
The diagrammatic depictions of our investigation. The IVW model refers to the inverse variance weighted model

This research made use of publicly accessible data. All original investigations received ethical clearance and informed permission from their subjects. To investigate the causal connection between ASD and 731 immune cell signatures (7 groups), we conducted two separate bi-directional, two-sample MR studies and a meta-analysis based on the two independent MR estimations. The MR technique involves three steps: (i) identification of instrumental variables (IVs) via the use of genetic variations; (ii) evaluation of causal estimations; and (iii) conducting sensitivity studies. Figure 1 displays the conceptual representations of our investigation.

### Immunity-wide GWAS data sources

The GWAS summary data for each immunological feature may be accessed publicly via the GWAS Catalog. The accession codes range from GCST90001391 to GCST90002121 (https://www.ebi.ac.uk/gwas/studies) [12]. A comprehensive set of 731 immunophenotypes was analyzed, which included several measurements such as absolute cell counts (*n* = 118), median fluorescence intensities (MFI) indicating surface antigen levels (*n* = 389), morphological parameters (MP) (*n* = 32), and relative cell counts (*n* = 192). More precisely, the MFI, AC, and RC characteristics include B cells, CDCs, fully developed T cells, monocytes, myeloid cells, TBNK (T cells, B cells, natural killer cells), and Treg panels. On the other hand, the MP characteristic includes CDC and TBNK panels. The first genome-wide association study (GWAS) on immunological variables included data from 3,757 people of European descent, with no cohorts overlapping. Around 22 million single nucleotide polymorphisms (SNPs) were genotyped using high-density arrays. These SNPs were imputed using a Sardinian sequence-based reference panel [13]. Associations were then assessed, taking into account variables such as sex, age, and age squared (Table 1).

**Table 1:** Baseline data table for exposure and outcome

Subject		Year	Population	Sample size	ID	Links for data download
Exposure	Immune cells	2020	Sardinians	3,757	PMID: 32,929,287	http://ftp.ebi.ac.uk/pub/databases/gwas/summary_statistics/
Outcome	ASD	2015	European	10,610	GWAS ID:ieu-a-1184	https://gwas.mrcieu.ac.uk/datasets/ieu-a-1184/
	ASD	2015	European	10,263	GWAS ID:ieu-a-806	https://gwas.mrcieu.ac.uk/datasets/ieu-a-806/

### GWAS data sources for ASD

We discovered a grand total of 7 ASD datasets published between the years 2013 and 2021 while doing a search for ASD datasets in the IEU OPEN GWAS PROJECT database. In order to prevent the issue of having duplicate inclusion samples, we opted for two datasets, namely ieu-a-1184 and ieu-a-806, which were both released in the same year. Simultaneously, we considered factors such as race, sample size, and the quantity of SNPs. The datasets were both published in 2015 and consist of individuals of European origin. The first dataset has a sample size of 10,610, while the second dataset has a sample size of 10,263. The SNPs identified were 9,499,589 and 9,499,590, respectively (Table 1).

### Selection of instrumental tools

A series of quality control measures were conducted to choose appropriate genetic instrumental instruments [14]. Based on current research [15, 16], we examined the single nucleotide polymorphisms (SNPs) that are linked to each immune trait at a significance level of *p* < 5 × 10^–5^, with a clumping window size greater than 10,000 kb and a linkage disequilibrium level of *r*^2^ < 0.001. In the case of ASD, we modified the significance threshold to 5 × 10^–8^. The F statistic was used to verify the robust correlation between IVs and exposure. A value of the F statistic over 10 was often deemed to satisfy the criteria for a strong connection [17].

### Statistical analysis

In this work, we conducted two separate bidirectional, two-sample MR studies. The research used four MR approaches to investigate the causal relationships between 731 immunophenotypes and ASD: inverse variance weighted (IVW), MR-Egger method, weighted model, and weighted median method. The IVW methodology was chosen as the primary method because it used a weighted linear regression model. The Cochran’s Q statistic and its accompanying P-values were used to assess the heterogeneity among the chosen IVs. In the event that the null hypothesis is rejected, the fixed-effects IVW method is substituted by the random-effects IVW method [18]. We used the MR-Egger regression intercept test to detect the presence of horizontal pleiotropy. In addition, the MR pleiotropy residual sum approach was used to eliminate any horizontal pleiotropic outliers that might significantly impact the estimate findings. Furthermore, scatter plots and funnel plots were used. Ultimately, we used a fixed-effect model to conduct a meta-analysis on the two MR estimations produced from the IVW-MRE method. The statistical analyses were conducted using the R software (version 4.0.3). The MR analysis was conducted using the TwoSampleMR programs [19]. The TwoSampleMR package enables the execution of Mendelian randomization using GWAS summary data. It automatically retrieves data from the IEU GWAS database and offers a wide range of methods for conducting the analysis. In order to tackle the problem of multiple testing, we used a Bonferroni-corrected significance criterion [20, 21]. This threshold was determined by dividing 0.05 by the total number of immunophenotypes (731), resulting in a value of 0.000068. P values ranging from 0.000068 to 0.05 were deemed to suggest potential causal relationships between the exposures and the outcomes. Causal connections between the exposures and the outcomes were judged significant if the P-value was less than 0.000068.

## Results

### Investigation into the causative impact of immunophenotypes on ASD

**Fig. 2:**
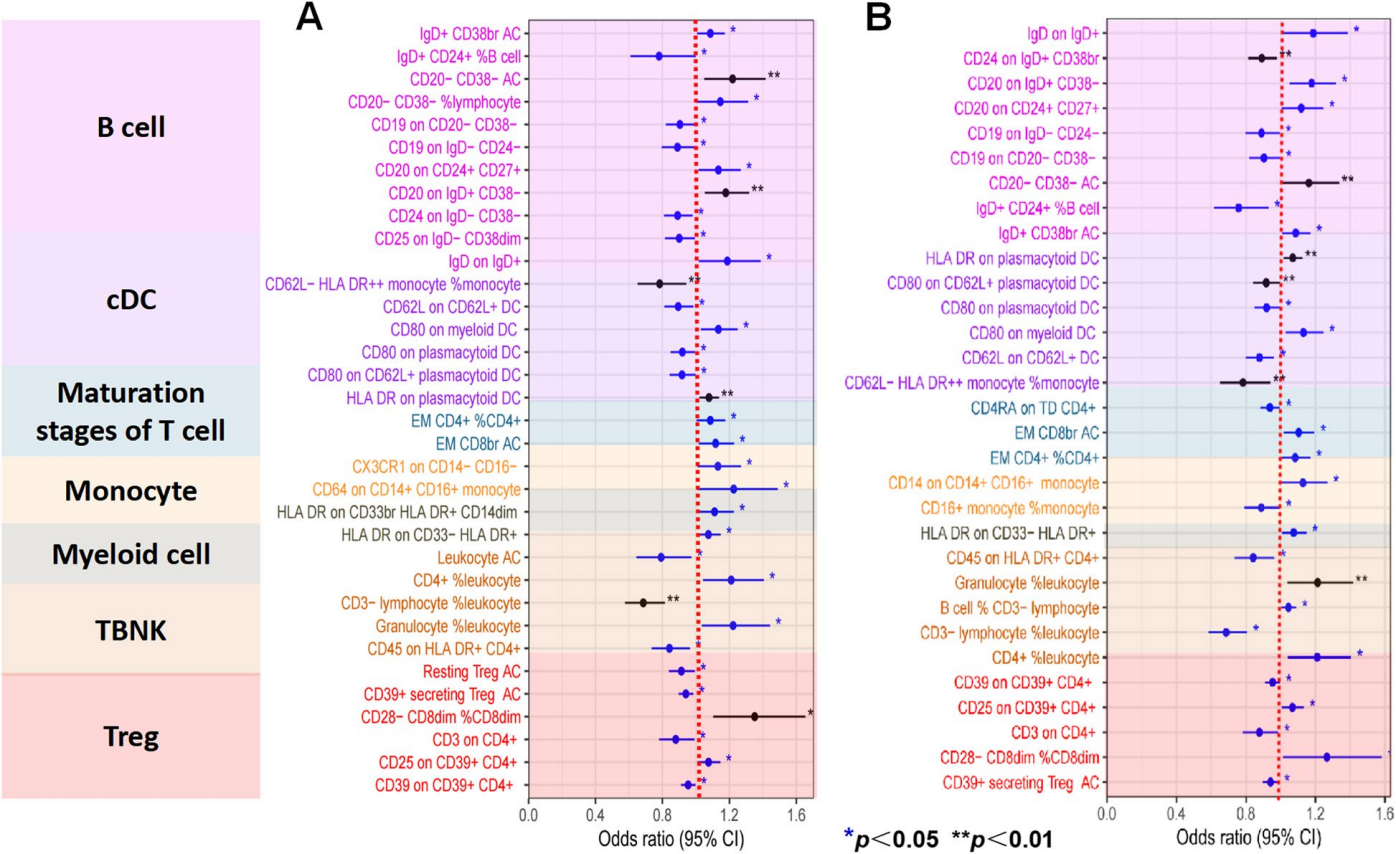
Forest plots illustrate the causal associations between immune cell characteristics and ASD by using IVW methods. **A** Estimates based on the ieu-a-1184 dataset; **B** Estimates based on the ieu-a-806 dataset

**Fig. 3:**
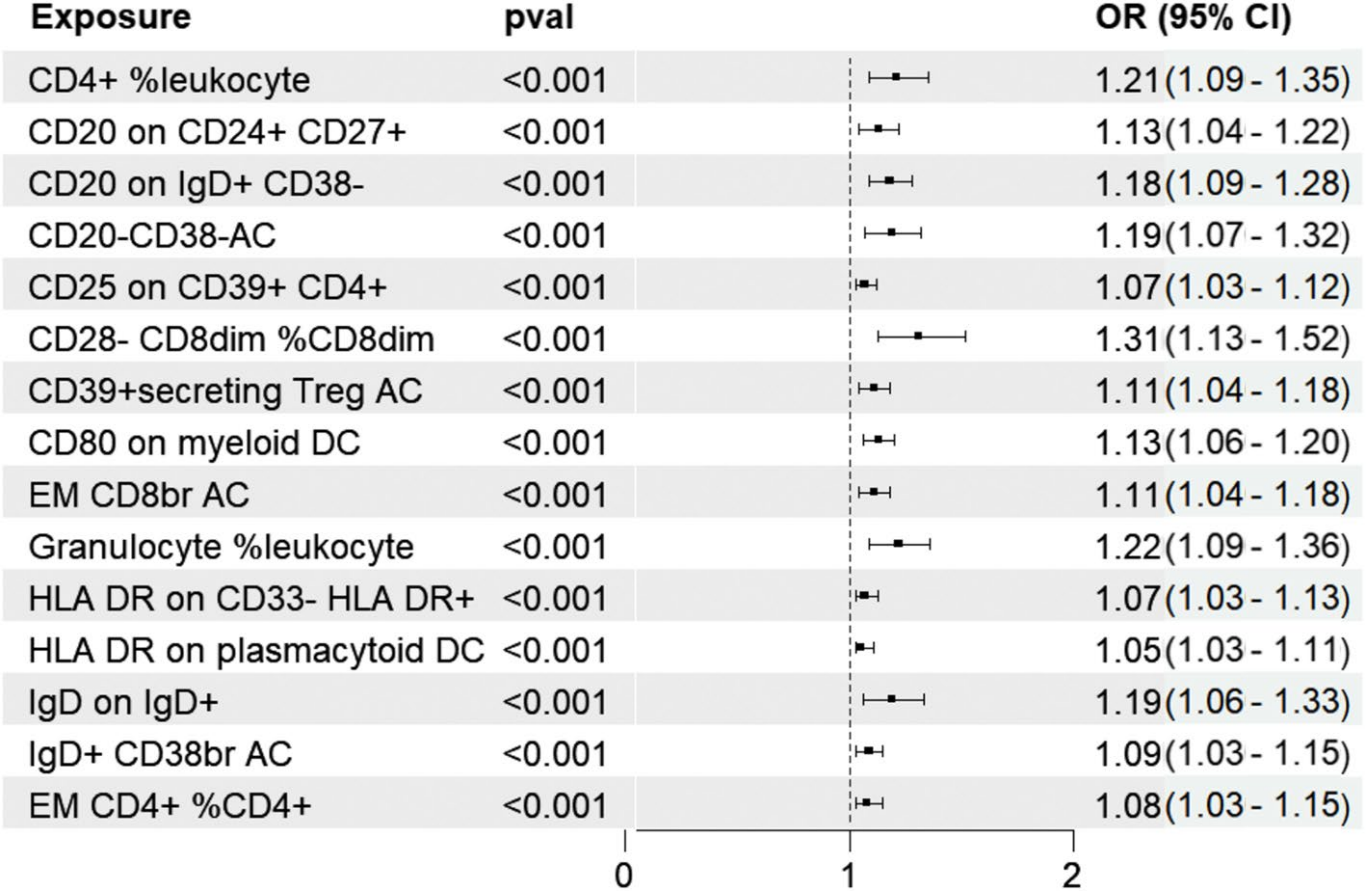
The meta-analysis of the two IVW-derived MR estimates revealed fifteen positive causal directions associated with ASD. The MR results are based on the GWAS of the ieu-a-1184 dataset and the ieu-a-806 dataset

**Fig. 4:**
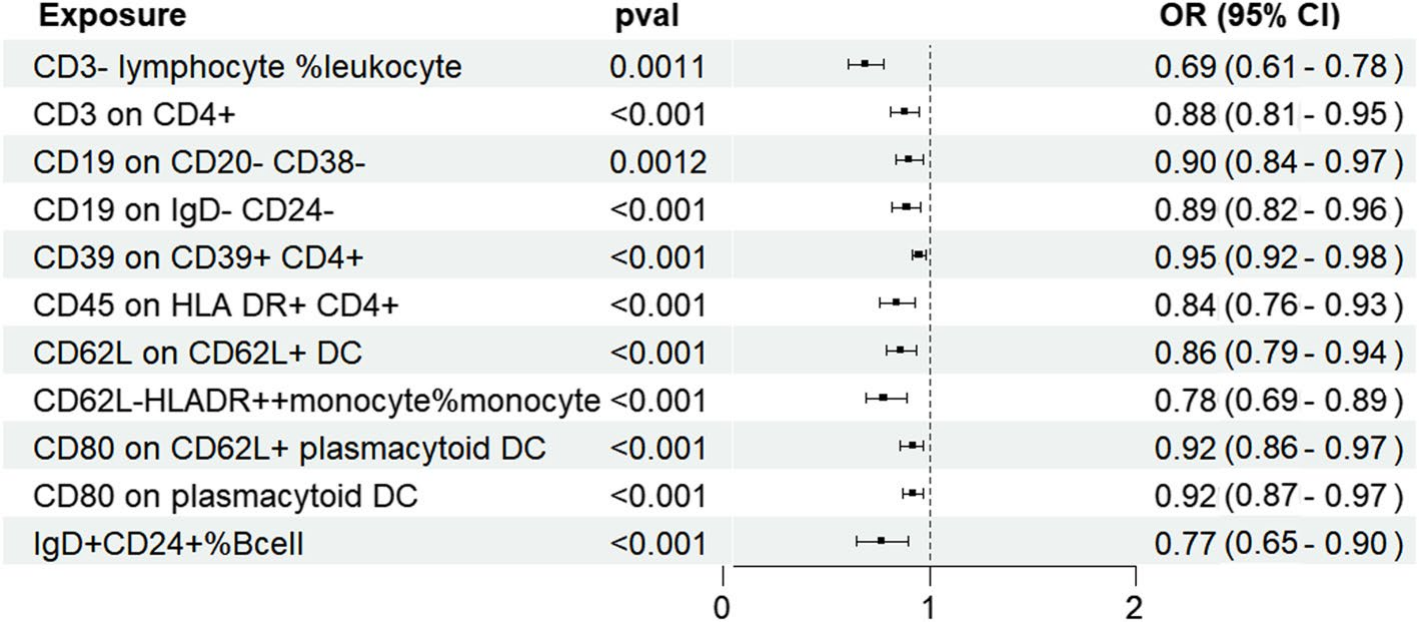
The meta-analysis of the two IVW-derived MR estimates revealed eleven reverse causal directions associated with ASD. The MR results are based on the GWAS of the ieu-a-1184 dataset and the ieu-a-806 dataset

IVW was selected as the principal MR method. According to the analysis of the ieu-a-1184 dataset, there are sixteen immunophenotypes that have a beneficial effect on ASD, whereas there are eighteen immunophenotypes that are associated with an elevated susceptibility to ASD (Fig. 2A). The results of the Bonferroni-adjusted analysis show a strong causal relationship between ASD and CD3- lymphocyte %leukocyte (*P* = 0.00002), additionally, there is a potential causal relationship between ASD and the other 33 immune phenotypes (Table 2). The analysis of the ieu-a-806 dataset revealed that fifteen immunophenotypes have a protective impact on ASD, whereas sixteen immunophenotypes are linked to an increased vulnerability to ASD (Fig. 2B). The Bonferroni-adjusted analysis indicates a substantial causative connection between ASD and CD3- lymphocyte%leukocyte (*P* = 0.00001). ASD may have a potential causal relationship with other 30 immune phenotypes (Table 3). Between the ieu-a-806 and ieu-a-1184 datasets, eleven potential positive causal directions with ASD and fifteen potential reverse causal directions with ASD overlapped. The meta-analysis of the two IVW derived MR estimations showed that for fifteen potential positive causal directions with ASD, The meta-analysis of the two IVW derived MR estimations showed that for fifteen potential positive causal directions with ASD, including CD4 + %leukocyte (OR = 1.21, 95%CI: 1.09–1.35, P < 0.001), CD20 on CD24 + CD27 + (OR = 1.13, 95%CI: 1.04–1.22, P < 0.001),CD20 on IgD + CD38-(OR = 1.18, 95%CI: 1.09–1.28, P < 0.001), CD20-CD38-AC (OR = 1.19, 95%CI: 1.07–1.32,P < 0.001), CD39 + CD4 + (OR = 1.07, 95%CI: 1.03–1.12,P < 0.001), CD28- CD8dim %CD8dim (OR = 1.31, 95%CI: 1.13–1.52,P < 0.001), CD39 + secreting Treg AC(OR = 1.11, 95%CI: 1.04–1.18,P < 0.001), CD80 on myeloid DC (OR = 1.13, 95%CI: 1.06–1.20,P < 0.001), EM CD8br AC(OR = 1.11, 95%CI: 1.04–1.18,P < 0.001), Granulocyte %leukocyte (OR = 1.22, 95%CI: 1.09–1.36,P < 0.001), HLA DR on CD33- HLA DR + (OR = 1.07, 95%CI: 1.03–1.13,P < 0.001), HLA DR on plasmacytoid DC (OR = 1.05, 95%CI: 1.03–1.11,P < 0.001), IgD on IgD + (OR = 1.19, 95%CI: 1.06–1.33,P < 0.001), IgD + CD38br AC (OR = 1.09, 95%CI: 1.03–1.15,P < 0.001), and EM CD4 + %CD4 + (OR = 1.08, 95%CI: 1.03–1.15,P < 0.001) (Fig. 3). The meta-analysis of the two IVW-generated MR estimates revealed that the OR for eleven reverse potential causal directions with ASD was 0.69 (95%CI:0.61–0.78, *P* = 0.0011) for CD3-lymphocyte%leukocyte, 0.88 (95%CI:0.81–0.95, P < 0.001) for CD3 on CD4 + , 0.9 (95%CI:0.84–0.97, *P* = 0.0012) for CD19 on CD20-CD38-, 0.89 (95%CI:0.82–0.96, P < 0.001) for CD19 on IgD- CD24-, 0.95 (95%CI:0.92–0.98, P < 0.001) for CD39 on CD39 + CD4 + , 0.84 (95%CI:0.76–0.93, P < 0.001) for CD45 on HLA DR + CD4 + , 0.86 (95%CI:0.79–0.94, P < 0.001) for CD62L on CD62L + DC, 0.78 (95%CI:0.69–0.89, P < 0.001) for CD62L-HLADR +  + monocyte% monocyte, 0.92 (95%CI:0.86–0.97, P < 0.001) for CD80 on CD62L + plasmacytoid DC, 0.92 (95% CI: 0.87–0.97, P < 0.001) on CD80 on plasmacytoid DC, and 0.77 (95% CI: 0.65–0.9, P < 0.001) for IgD + CD24 + %Bcell (Fig. 4). For all MR analyses, sensitivity tests revealed no indication of effect size heterogeneity (leave-one-out; Cochran Q statistic, *P* > 0.10) or pleiotropy (MR-Egger intercept, *P* > 0.05) (Supplementary Table 2,3,5,and 6). Scatter plots and funnel plots also confirmed the findings’ stability (Supplementary Fig. 1–4).

**Table 2:** MR estimations of the immunophenotypes’s causal role on ASD( ieu-a-1184 datasets)

Panel	Trait	Method	*P*val	OR(95%CI)
**B cell**	IgD + CD38br AC	IVW	0.03282	1.08635(1.00678,1.17219)
	IgD + CD24 + %B cell	IVW	0.04939	0.77986(0.60859,0.99934)
	CD20- CD38- AC	IVW	0.00964	1.21931(1.04932,1.41685)
	CD20- CD38- %lymphocyte	IVW	0.04874	1.14598(1.00075,1.31229)
	CD19 on CD20- CD38-	IVW	0.04465	0.90434(0.81979,0.99760)
	CD19 on IgD- CD24-	IVW	0.04051	0.88990(0.79593,0.99497)
	CD20 on CD24 + CD27 +	IVW	0.02621	1.13452(1.01504,1.26806)
	CD20 on IgD + CD38-	IVW	0.00423	1.17819(1.05297,1.31831)
	CD24 on IgD- CD38-	IVW	0.01807	0.89063(0.80908,0.98039)
	CD25 on IgD- CD38dim	IVW	0.03808	0.90025(0.81514,0.99424)
	IgD on IgD +	IVW	0.03004	1.18762(1.01674,1.38721)
**cDC**	CD62L- HLA DR + + monocyte %monocyte	IVW	0.00965	0.78350(0.65133,0.94250)
	CD62L on CD62L + DC	IVW	0.02508	0.89432(0.81106,0.98613)
	CD80 on myeloid DC	IVW	0.01094	1.13424(1.02937,1.24979)
	CD80 on plasmacytoid DC	IVW	0.03853	0.91915(0.84861,0.99556)
	CD80 on CD62L + plasmacytoid DC	IVW	0.04498	0.91709(0.84269,0.99807)
	HLA DR on plasmacytoid DC	IVW	0.00637	1.07819(1.02143,1.13812)
**Maturation stages of T cell**	EM CD4 + %CD4 +	IVW	0.04076	1.08617(1.00348,1.17567)
	EM CD8br AC	IVW	0.02089	1.11760(1.01699,1.22815)
**Monocyte**	CX3CR1 on CD14- CD16-	IVW	0.03549	1.13152(1.00842,1.26964)
	CD64 on CD14 + CD16 + monocyte	IVW	0.04086	1.22553(1.00849,1.48929)
**Myeloid cell**	HLA DR on CD33br HLA DR + CD14dim	IVW	0.03514	1.11157(1.00740,1.22652)
	HLA DR on CD33- HLA DR +	IVW	0.03310	1.07465(1.00579,1.14823)
**TBNK**	Leukocyte AC	IVW	0.02679	0.79194(0.64421,0.97355)
	CD4 + %leukocyte	IVW	0.01238	1.21064(1.04223,1.40626)
	CD3- lymphocyte %leukocyte	IVW	0.00002	0.68569(0.57682,0.81512)
	Granulocyte %leukocyte	IVW	0.01797	1.22223(1.03505,1.44326)
	CD45 on HLA DR + CD4 +	IVW	0.01312	0.84188(0.73482,0.96453)
**Treg**	Resting Treg AC	IVW	0.03620	0.91307(0.83859,0.99417)
	CD39 + secreting Treg AC	IVW	0.01045	0.94002(0.89654,0.98560)
	CD28- CD8dim %CD8dim	IVW	0.00359	1.35111(1.10339,1.65445)
	CD3 on CD4 +	IVW	0.03571	0.87691(0.78036,0.99147)
	CD25 on CD39 + CD4 +	IVW	0.02505	1.07557(1.00917,1.14634)
	CD39 on CD39 + CD4 +	IVW	0.04118	0.95376(0.91138,0.99811)

**Table 3:** MR estimations of the immunophenotypes’s causal role on ASD ( ieu-a-806 datasets)

Panel	Trait	Method	*P*val	OR(95%CI)
**B cell**	IgD on IgD +	IVW	0.03004	1.18762(1.06174,1.38721)
	CD24 on IgD + CD38br	IVW	0.01542	0.89218(0.81351,0.97846)
	CD20 on IgD + CD38-	IVW	0.00423	1.17819(1.05297,1.31831)
	CD20 on CD24 + CD27 +	IVW	0.04194	1.11835(1.00408,1.24561)
	CD19 on IgD- CD24-	IVW	0.04051	0.89990(0.79593,0.99497)
	CD19 on CD20- CD38-	IVW	0.04465	0.90434(0.81979,0.99760)
	CD20- CD38- AC	IVW	0.03462	1.16236(1.01094,1.33646)
	IgD + CD24 + %B cell	IVW	0.00829	0.75782(0.61680,0.93107)
	IgD + CD38br AC	IVW	0.03282	1.08635(1.00678,1.17219)
**cDC**	HLA DR on plasmacytoid DC	IVW	0.00914	1.06958(1.01683,1.12507)
	CD80 on plasmacytoid DC	IVW	0.03853	0.91915(0.84861,0.99556)
	CD80 on CD62L + plasmacytoid DC	IVW	0.04498	0.91709(0.84269,0.99807)
	CD80 on myeloid DC	IVW	0.01222	1.13135(1.02725,1.24601)
	CD62L on CD62L + DC	IVW	0.00573	0.87779(0.80027,0.96283)
	CD62L- HLA DR + + monocyte %monocyte	IVW	0.00965	0.78350(0.65133,0.94250)
**Maturation stages of T cell**	CD4RA on TD CD4 +	IVW	0.03964	0.93827(0.88302,0.99699)
	EM CD8br AC	IVW	0.01500	1.10392(1.01939,1.19546)
	EM CD4 + %CD4 +	IVW	0.04384	1.08372(1.00222,1.17184)
**Monocyte**	CD14 on CD14 + CD16 + monocyte	IVW	0.04273	1.12879(1.00397,1.26913)
	CD16 + monocyte %monocyte	IVW	0.04466	0.88804(0.79085,0.99717)
**Myeloid cell**	HLA DR on CD33- HLA DR +	IVW	0.03310	1.07465(1.00579,1.14823)
**TBNK**	CD45 on HLA DR + CD4 +	IVW	0.01312	0.84188(0.73482,0.96453)
	Granulocyte %leukocyte	IVW	0.01424	1.21315(1.03947,1.41584)
	B cell % CD3- lymphocyte	IVW	0.03889	1.04496(1.00224,1.08950)
	CD3- lymphocyte %leukocyte	IVW	0.00001	0.68672(0.58363,0.80801)
	CD4 + %leukocyte	IVW	0.01238	1.21064(1.04223,1.40626)
**Treg**	CD39 on CD39 + CD4 +	IVW	0.03883	0.95332(0.91106,0.99755)
	CD25 on CD39 + CD4 +	IVW	0.03400	1.06744(1.04494,1.13382)
	CD3 on CD4 +	IVW	0.02884	0.87793(0.78120,0.98665)
	CD28- CD8dim %CD8dim	IVW	0.03827	1.26648(1.01285,1.58362)
	CD39 + secreting Treg AC	IVW	0.01249	0.94154(0.89808,0.98711)

### Investigation into the causative impact of ASD development on immunophenotypes

**Fig. 5:**
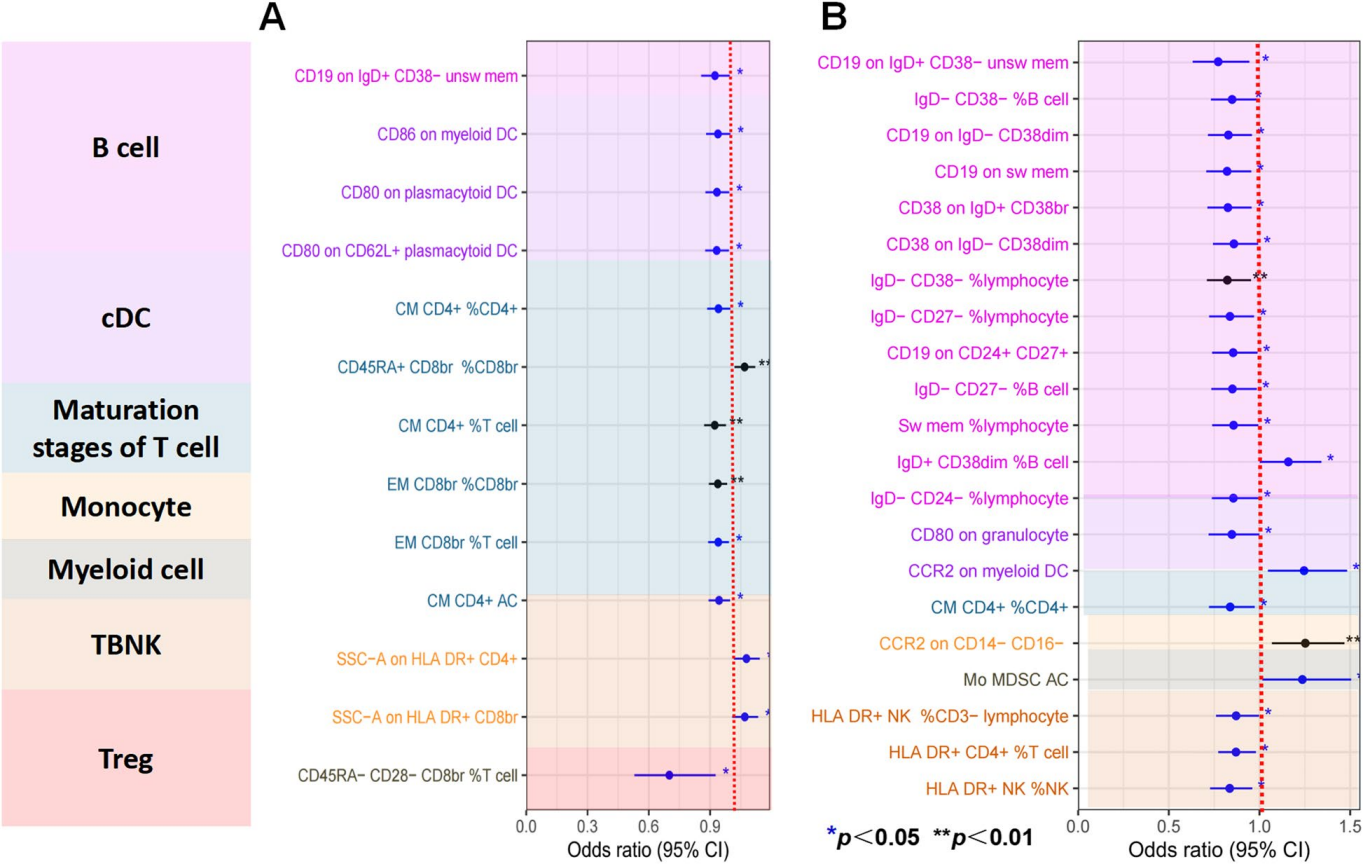
shows the causal associations between ASD and immune cell characteristics by using the IVW method. **A** Estimates based on the ieu-a-805 dataset; **B** Estimates based on the ieu-a-1148 dataset

**Fig. 6:**
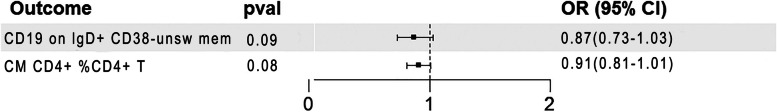
Meta-analysis estimates of MR results from the IVW model based on the GWAS of the

In order to investigate the direct impacts of ASD on immunophenotypes, a two-sample MR study was conducted, with the IVW method used as the primary analytical approach. Through the GWAS of ieu-a-1184, the results of the Bonferroni-adjusted analysis revealed 13 immunophenotypes with potential indications (Fig. 5A and Table 4). GWAS analysis of ieu-a-806 identified 21 immunophenotypes with potential correlations based on Bonferroni-adjusted analysis(Fig. 5B and Table 5). By identifying the common features between the two groups, we discovered a potential causal relationship between a greater risk of ASD and a lower level of circulating CD19 on IgD + CD38-unswitched memory B cells and CM CD4 + %CD4 + T cells. The meta-analysis of the two IVW-derived MR estimates resulted in an o OR of 0.87 (95% [CI] 0.73–1.03, *P* = 0.09) for CD19 on IgD + CD38-unswitched memory B cells and 0.91 (95% [CI] 0.81–1.01, *P* = 0.08) for CM CD4 + %CD4 + T cells (Fig. 6).

**Table 4:** MR estimations of the ASD’s causal role on immunophenotypes ( ieu-a-1184 datasets)

Panel	Trait	Method	*P*val	OR(95%CI)
**B cell**	CD19 on IgD + CD38- unsw mem	IVW	0.04103	0.92379 (0.85614,0.99678)
**cDC**	CD86 on myeloid DC	IVW	0.04964	0.93839 (0.88066,0.99990)
	CD80 on plasmacytoid DC	IVW	0.03189	0.93366 (0.87691,0.99407)
	CD80 on CD62L + plasmacytoid DC	IVW	0.02851	0.93233 (0.87566,0.99266)
**Maturation stages of T cell**	CM CD4 + %CD4 +	IVW	0.04835	0.94081 (0.88552,0.99956)
	CD45RA + CD8br %CD8br	IVW	0.00618	1.06885 (1.01911,1.12103)
	CM CD4 + %T cell	IVW	0.00677	0.92268 (0.87047,0.97802)
	EM CD8br %CD8br	IVW	0.00806	0.93781 (0.89431,0.98343)
	EM CD8br %T cell	IVW	0.02539	0.93994 (0.89026,0.99240)
	CM CD4 + AC	IVW	0.04375	0.94390 (0.89238,0.99838)
**TBNK**	SSC-A on HLA DR + CD4 +	IVW	0.01225	1.07794 (1.01646,1.14315)
	SSC-A on HLA DR + CD8br	IVW	0.02528	1.06983 (1.00840,1.13501)
**Treg**	CD45RA- CD28- CD8br %T cell	IVW	0.01284	0.70061 (0.52936,0.92725)

**Table 5:** MR estimations of the ASD’s causal role on immunophenotypes ( ieu-a-806 datasets)

Panel	Trait	Method	*P*val	OR(95%CI)
**B cell**	IgD- CD38- %B cell	IVW	0.02958	0.84881 (0.73227, 0.98388)
	CD19 on IgD + CD38- unsw mem	IVW	0.01173	0.77207 (0.63137, 0.94413)
	CD19 on IgD- CD38dim	IVW	0.01139	0.82837 (0.71595, 0.95844)
	CD19 on sw mem	IVW	0.01043	0.82116 (0.70622, 0.95480)
	CD38 on IgD + CD38br	IVW	0.01066	0.82563 (0.71271, 0.95644)
	CD38 on IgD- CD38dim	IVW	0.03923	0.85879 (0.74309, 0.99252)
	IgD- CD38- %lymphocyte	IVW	0.00946	0.82262 (0.70982, 0.95335)
	IgD- CD27- %lymphocyte	IVW	0.01801	0.83695 (0.72219, 0.96995)
	CD19 on CD24 + CD27 +	IVW	0.03542	0.85468 (0.73835, 0.98934)
	IgD- CD27- %B cell	IVW	0.03088	0.85053 (0.73425, 0.98521)
	Sw mem %lymphocyte	IVW	0.04126	0.85700 (0.73896, 0.99390)
	IgD + CD38dim %B cell	IVW	0.04842	1.15879 (1.00102, 1.34142)
	IgD- CD24- %lymphocyte	IVW	0.04076	0.85585 (0.73728, 0.99348)
**cDC**	CD80 on granulocyte	IVW	0.04878	0.84769 (0.71922, 0.9912)
	CCR2 on myeloid DC	IVW	0.01365	1.24551 (1.04610, 1.48284)
**Maturation stages of T cell**	CM CD4 + %CD4 +	IVW	0.02083	0.83815 (0.72160, 0.97353)
**Monocyte**	CCR2 on CD14- CD16-	IVW	0.00556	1.25244 (1.06820, 1.46846)
**Myeloid cell**	Mo MDSC AC	IVW	0.03491	1.23628 (1.01514, 1.50561)
**TBNK**	HLA DR + NK %CD3- lymphocyte	IVW	0.04634	0.87104 (0.76041, 0.99776)
**TBNK**	HLA DR + CD4 + %T cell	IVW	0.02228	0.87024 (0.77247, 0.98039)
**TBNK**	HLA DR + NK %NK	IVW	0.01108	0.83565 (0.72755, 0.95982)

## Discussion

Numerous efforts have been made to establish the etiology of ASD; nonetheless, it remains mainly mysterious. The development of ASD is believed to be influenced by genetic, neurological, immunological, and environmental factors. Neuroimmunology is receiving increasing focus due to the potential connection between immune response dysregulation and abnormalities in neurodevelopment. Multiple studies have shown altered immune system function in patients with ASD [22]. In this study, we used a bi-directional, two-sample Mendelian randomization method to establish that 26 specific immunophenotypes had potential causal effects on ASD across four distinct immunological characteristics (MFI, RC, AC, and MP). This work is the first effort to uncover the cause-and-effect connection between 731 immune cell characteristics and ASD using a genetic method that relies on GWA summary data.

T-lymphocytes have a crucial function in facilitating adaptive immunity through the secretion of cytokines, which help regulate immune responses at the local level [23]. T-lymphocytes are comprised of two subsets: CD8 cytotoxic T cells and CD4 helper T cells. CD4 T cells that have been activated undergo cellular division and transform into distinct subsets of T-helper (Th) cells. The subtypes mentioned are Th1, Th2, Th17, and regulatory T (Treg) cells [24]. Treg cells have a role in avoiding the occurrence of autoimmune diseases and maintaining the body’s ability to tolerate itself by releasing the anti-inflammatory cytokines IL-10 and TGF-β [25]. IL-10 is produced by Th2 and Treg cells, along with various other immune cells, in order to restrict the excessive release of pro-inflammatory cytokines and chemokines during infection and reduce tissue damage [26]. Our study discovered that there is a negative correlation between the risk of ASD and the proportion of CD3 on CD4 + regulatory T cells decreases. An analysis of immune cells in the blood of individuals with ASD has shown a significant decrease in Treg compared to healthy individuals, as demonstrated by De Giacomo et al. in 2021 [27]. Furthermore, a considerable proportion of individuals with ASD have impairments in CD4 + and CD25 + Tregs, resulting in the activation of the immune system against self-tissues in a specific subset of these patients [28]. In addition, a recent meta-analysis has shown significant abnormalities in CD4 + lymphocytes, namely a decrease in Tregs and an increase in Th17 cells, in individuals with ASD [27]. Treg cells have a crucial function in maintaining self-tolerance by restraining autoimmune reactions via the suppression of proinflammatory mechanisms [29]. Several investigations have shown abnormalities in lymphocytes and imbalances among different types of lymphocyte subgroups. The most consistent results in individuals with ASD include reduced responsiveness to stimulation [30, 31], aberrant activation [32], an imbalanced ratio of T helper and suppressor cells [33], decreased activity of Th cells [34], and a systemic deficiency of Tregs [35]. The findings as a whole indicate that a significant percentage of patients with ASD have changes in the function of their lymphocytes, particularly in T-cell subpopulations. These findings, in conjunction with cytokine irregularities, provide a more comprehensive perspective on a potential underlying cause for the reported anomalies in ASD.

Natural killer (NK) cells make up around 15% of the lymphocytes found in the bloodstream and have a crucial function in the innate immune system [36]. These cells are distinguished by the absence of CD3 surface antigen and the presence of CD56, and their activity is carried out by the secretion of immunomodulatory cytokines like IFN-γ, tumor necrosis factor-alpha (TNF-α), and IL-10. In addition, they possess cytolytic activity and facilitate cellular cytotoxicity and immunological surveillance by interacting with dendritic cells [37, 38]. Discrepancies in the activation and inhibition states of some factors may contribute to the development of autoimmune disorders. However, the precise processes responsible for this phenomenon have not been completely elucidated [39]. NK cells have been implicated in the development of neurological illnesses such as multiple sclerosis [40], schizophrenia [41], Tourette syndrome [42], and Rett syndrome [43]. The impact of NK cells on ADS is a subject of debate in research. Two prior studies [44, 45] found that autistic people had larger absolute numbers of NK cells in their peripheral blood. While another study found that a large number of people with ASD had a decrease in their NK cell count [46], our study discovered that increasing the fraction of CD3-lymphocytes reduced the incidence of ASD. The selection and quantification of NK cells were based only on the use of CD56 and CD3 markers. CD56 and CD57 are markers that can be utilized to categorize many discrete subsets of NK cells. The transcriptional signature, phenotypic characteristics, and functional abilities of human CD57 + NK cells differ from those of CD57 + -NK cells [47, 48]. Siniscalco et al. discovered a decline in CD57 + CD3-numbers among individuals with ASD, although D56 + CD3-counts remained unchanged but within the normal range [46]. This suggests that a certain group of non-T lymphocytes with NK activity may play a crucial role in ASD.

A correlation between certain alleles of human leukocyte antigens (HLA) and autoimmune disorders has been discovered. Multiple studies have established connections between HLA and ASD, demonstrating that autistic children have a greater occurrence of the HLA-DRB1*11 allele and a reduced occurrence of the HLA-DRB1*03 allele [49]. Multiple researchers have documented a correlation between HLA and ASD in various populations, such as Caucasians [50] and Chinese [51]. Guerini et al. discovered an intriguing correlation between HLA-G polymorphism and ASD, which is likely due to prenatal immunological activation [52]. The presence of HLA-DR4 in mothers has been identified as a risk factor for ASD in their children [53]. When investigating the immunopathology of the disease, it is recommended to take into account genetic variations in the HLA region [54]. Our MR analysis indicated a potential causal association between HLA DR expression on plasmacytoid dendritic cells, the percentage of monocytes expressing CD62L-HLA DR +  + , HLA DR expression on CD33-HLA DR + cells, and CD45 expression on HLA DR + CD4 + TBNK cells, and ASD. This demonstrates the importance of HLA in the pathophysiology of ASD.

In individuals with ASD, there is an occurrence of abnormal numbers or activation of certain innate immune cells. This includes a notable increase in the quantity of peripheral myeloid dendritic cells (DCs) [55]. The alterations in the DC population might be attributed to an augmented process of monocyte differentiation into dendritic cells. Additional research has shown that peripheral myeloid DCs obtained from children with ASD have a higher level of surface CD80/CD86 co-stimulatory molecules compared to typically developing children [56]. DCs variations were shown to be associated with differences in amygdala volume, gastrointestinal problems, and impaired behaviors [55]. Our study found that the presence of CD80 on myeloid DC and HLA DR on plasmacytoid DC is associated with an increased risk of ASD. Conversely, the presence of CD62L on CD62L + DC, CD80 on plasmacytoid DC, and CD80 on CD62L + plasmacytoid DC is associated with a decreased risk of ASD. This indicates that different subtypes of dendritic cells have differing effects on the risk of ASD.

Heuer et al. examined the roles of B cells and noted a reduction in the overall levels of both IgM and IgG in the peripheral blood of children with ASD, as compared to typically developing individuals [57]. They subsequently examine whether the decreased amounts of IgG and IgM in the plasma were caused by impaired formation, activation, or activity of B cells. Heuer et al. demonstrated that there were no discernible disparities in the quantity of B memory cells. These findings suggest that the reduction of immunoglobulins (IgG) in individuals with ASD is not caused by B cell malfunction but rather is influenced by the participation of many immune cells [58]. Furthermore, based on a meta-analysis comprising five investigations, no statistically significant variations in B cell counts were seen between persons with ASD and normally developing controls [59]. Our findings indicate the presence of eight distinct B cell subtypes, with five showing a positive causal relationship and three showing a reverse causal association with ASD. Additional population-based and experimental studies are necessary to fully elucidate the intricate connection between specific subtypes of B lymphocytes and ASD in the future.

Naturally, this study has certain constraints. Initially, there is a possibility of selection bias due to the fact that the immune cell samples were mostly from individuals of Sardinian descent, whereas the samples of individuals with ASD were exclusively from European ethnicities. The Sardinian population, used for the GWAS of immune cell features, is genetically distinct as a result of its geographical isolation from mainland Europeans. Orru et al. (2020) discovered that out of the 122 genetic signals related to immune cell features, 16 (13%) had low frequencies among Europeans from the 1000 Genome Project, but high rates among Sardinians [12]. Furthermore, ASD is predominantly diagnosed in males, with a male-to-female ratio of up to 4:1. Our study did not distinguish between genders. In order to accurately interpret our findings, it is imperative to assess potential disparities between the outcomes when applied only to male or female populations.

In conclusion, we thoroughly investigated the causal relationship between the 731 immunophenotype and ASD. Our results suggest that there are eleven potential positive causal directions and fifteen potential reverse causal directions for ASD. The study indicates that specific types of immune cells and genetic predispositions could serve as biomarkers for the likelihood of developing ASD. This discovery has the potential to enable earlier detection and more efficient treatment strategies.

### Supplementary Information

Supplementary Material 1.

Supplementary Material 2.

Supplementary Material 3.

Supplementary Material 4.

Supplementary Material 5.

Supplementary Material 6.

Supplementary Material 7.

Supplementary Material 8.

Supplementary Material 9.

Supplementary Material 10.

Supplementary Material 11.

Supplementary Material 12.

Supplementary Material 13.

Supplementary Material 14.

Supplementary Material 15.

Supplementary Material 16.

## Data Availability

The research includes the original contributions, which may be found in the article/Supplementary material. For any more enquiries, please contact the corresponding author. GWAS data for ASD can be downloaded from the IEU-OpenGWAS project (https://gwas.mrcieu.ac.uk/). The GWAS Catalogue accession numbers are ieu-a-1184 and ieu-a-806. GWAS data for immune cells can be downloaded from the GWAS Catalogue (https://www.ebi.ac.uk/gwas/studies), with accession codes ranging from GCST90001391 to GCST90002121.
