# Supplementary material for: The relationship of immune cells with autism spectrum disorder: a bidirectional Mendelian randomization study
Source: BMC Psychiatry. 2024 Jun 27;24:477. doi: 10.1186/s12888-024-05927-5 (PMC11212275; doi:10.1186/s12888-024-05927-5)
Supplement: Supplementary file 1 — Supplementary Material 1. [file 12888_2024_5927_MOESM1_ESM.docx]

**Supplementary Figures**

A B C D


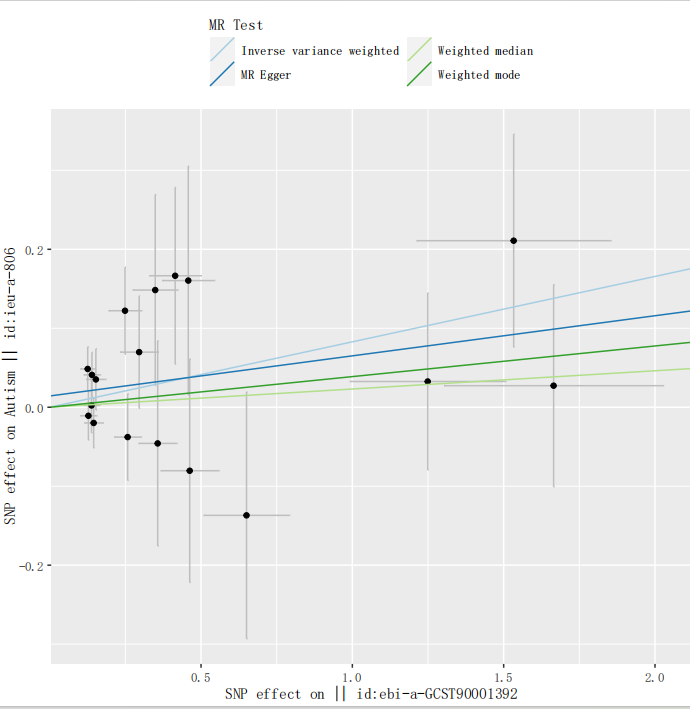

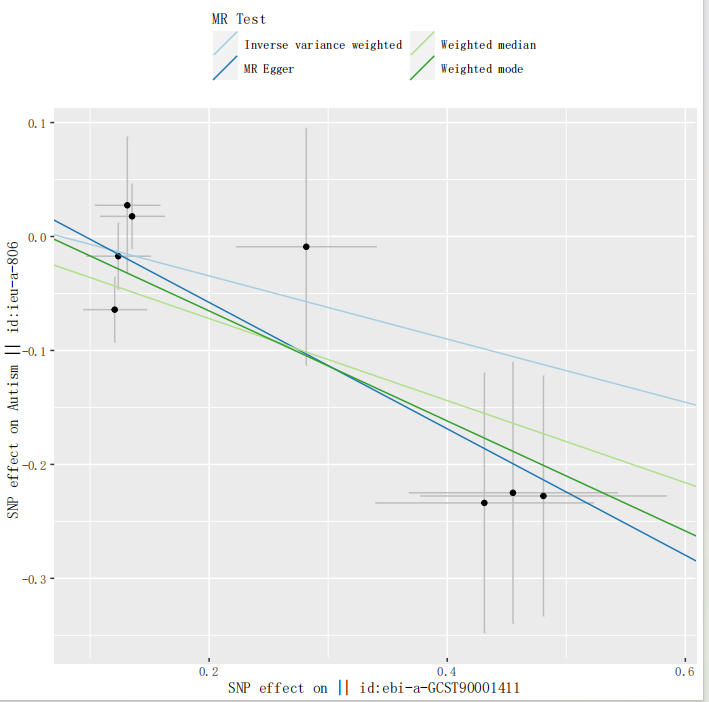

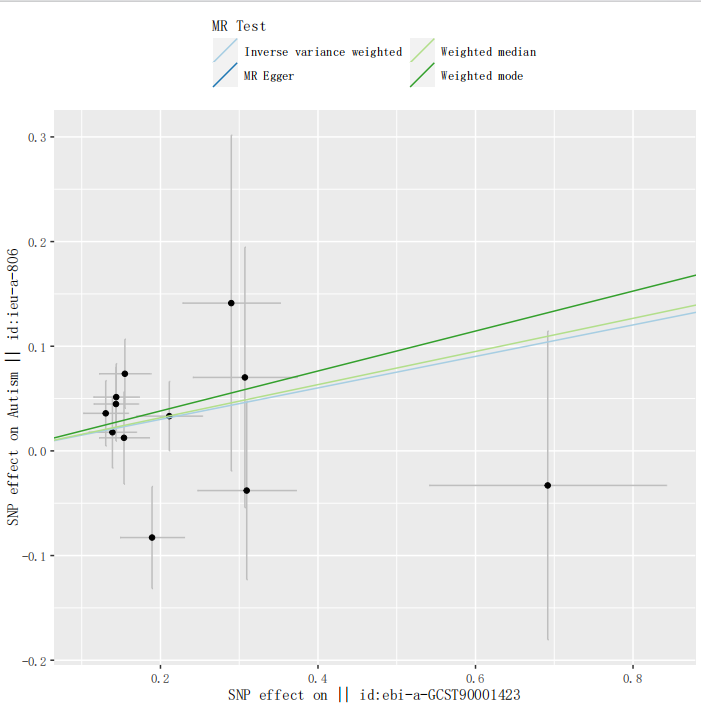

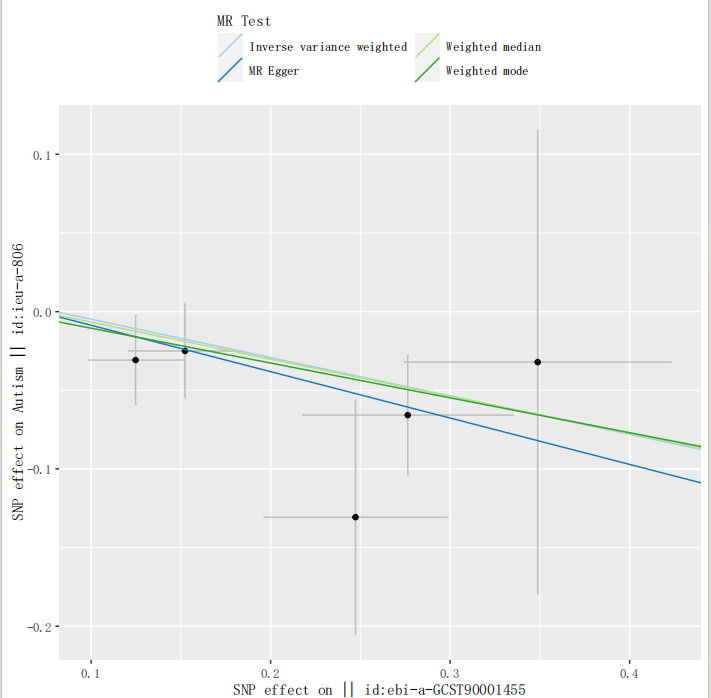


E F G H


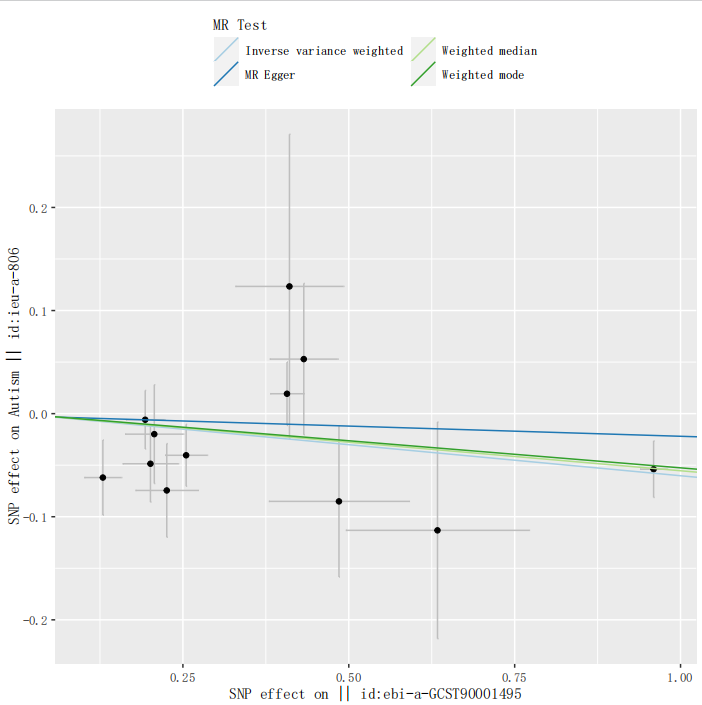

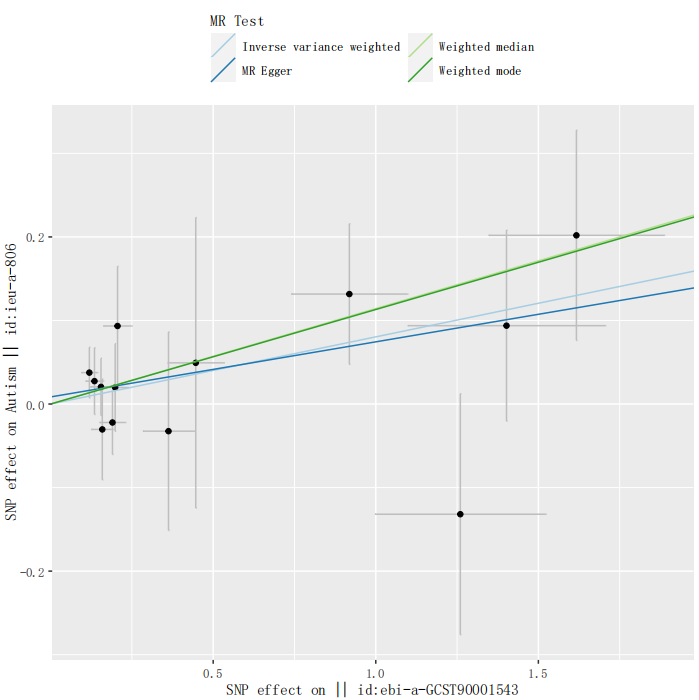

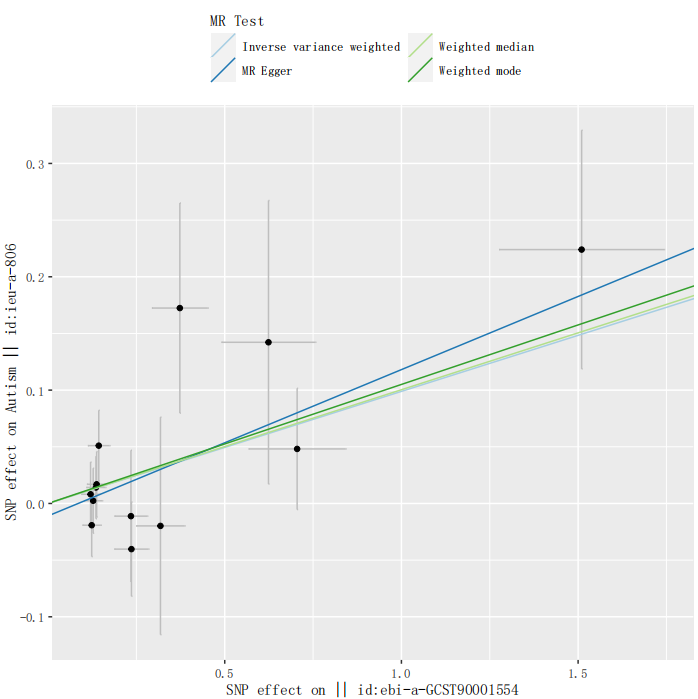

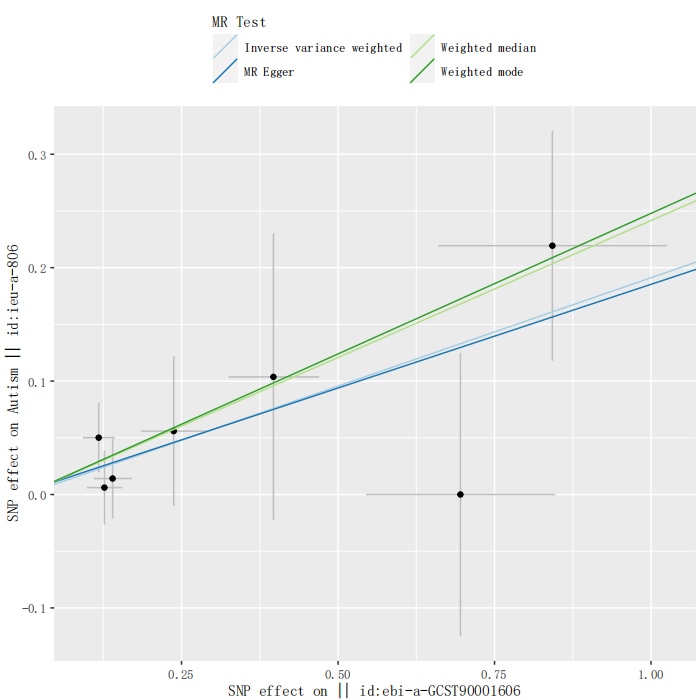


I J K L


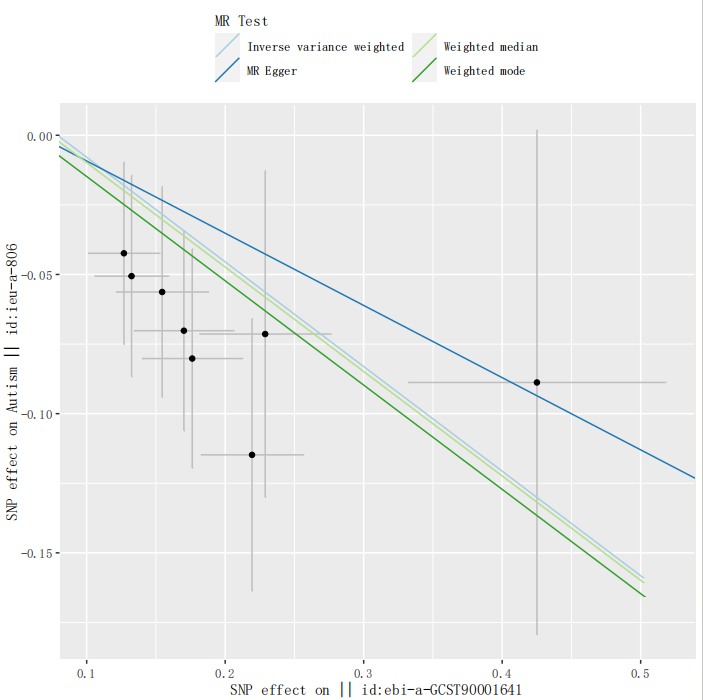

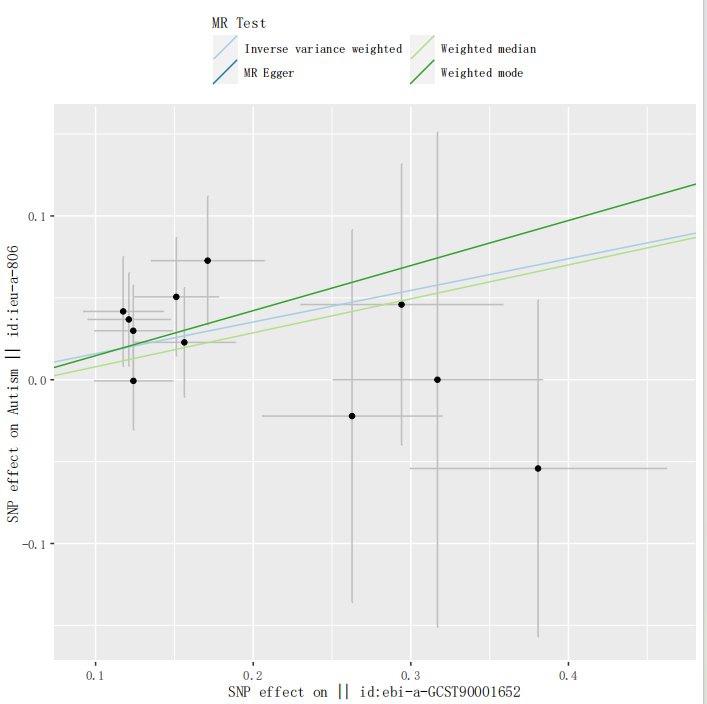

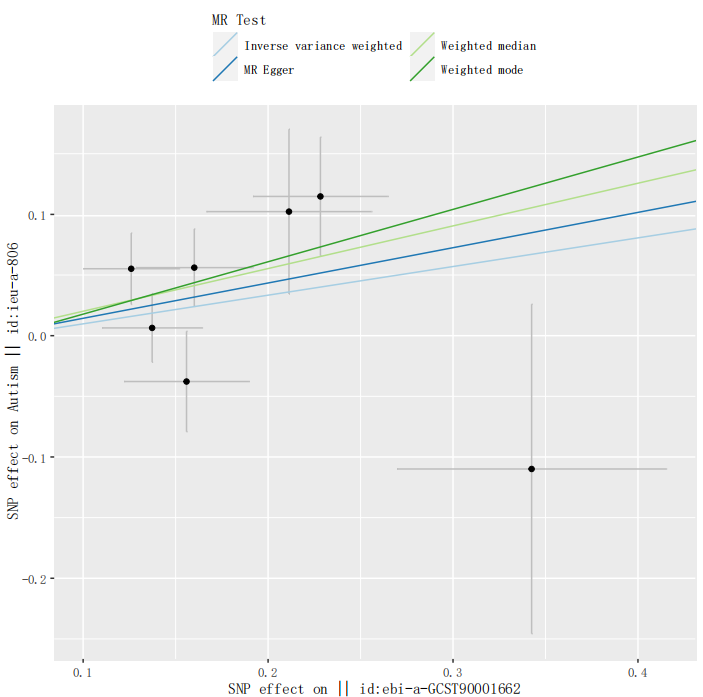

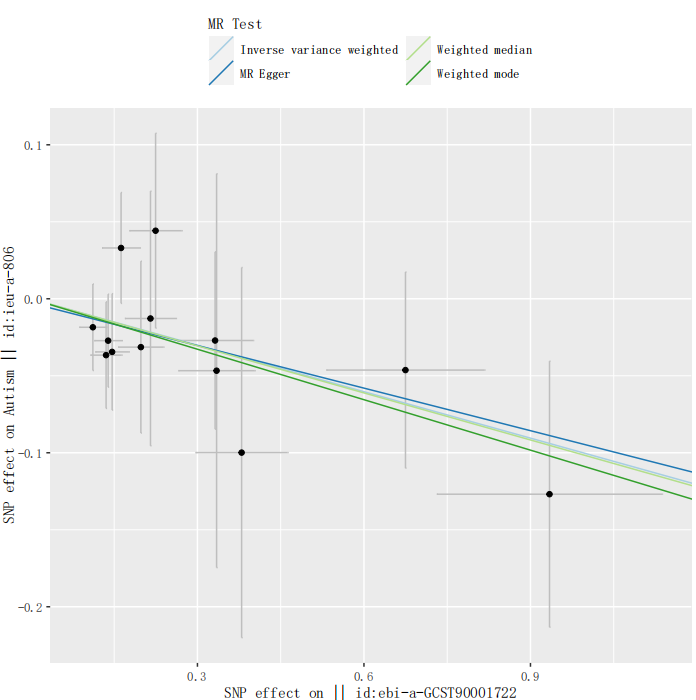


M N O P


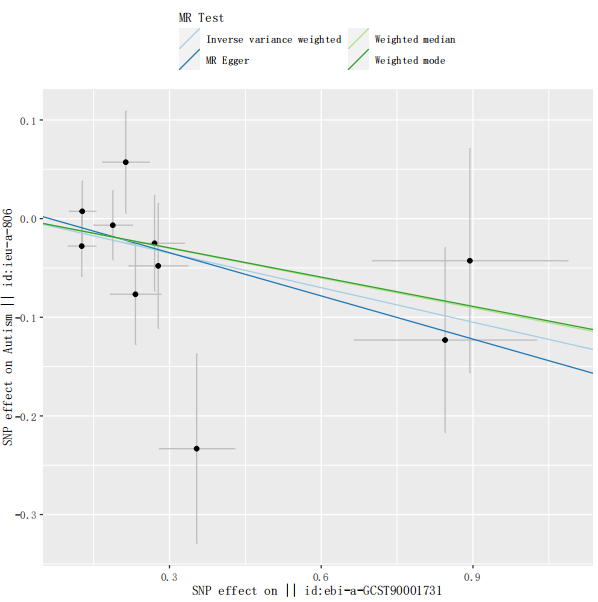

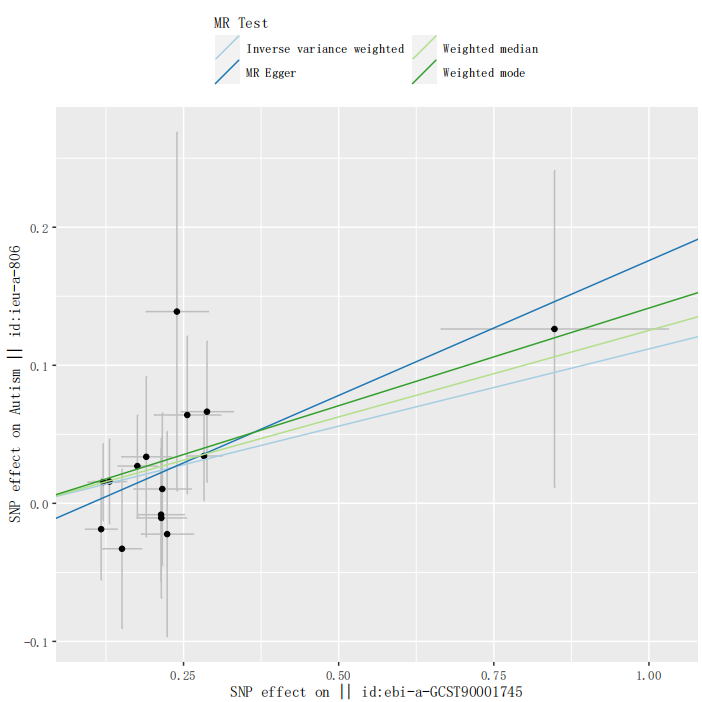

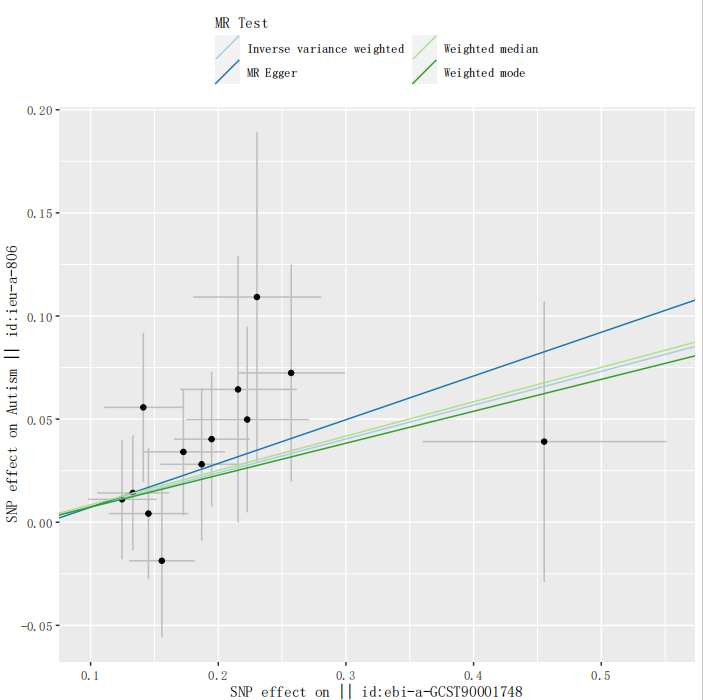

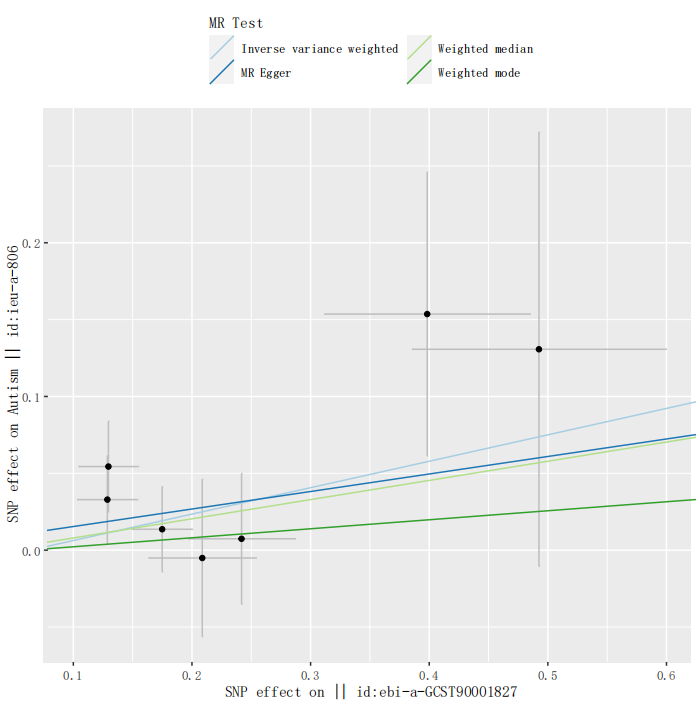


Q R S T


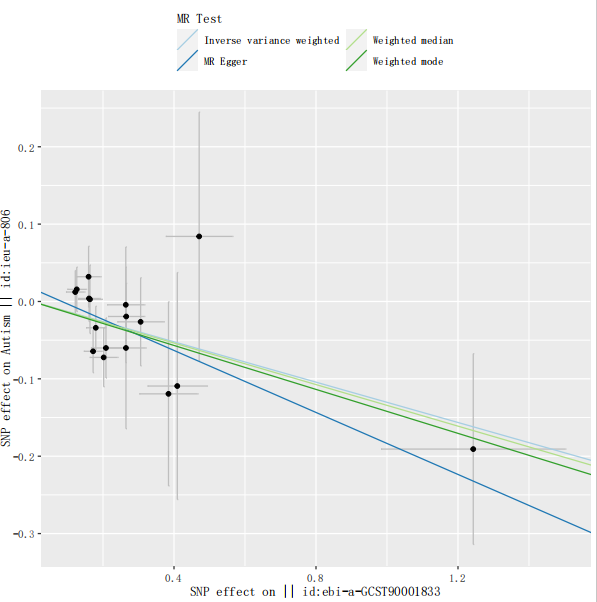

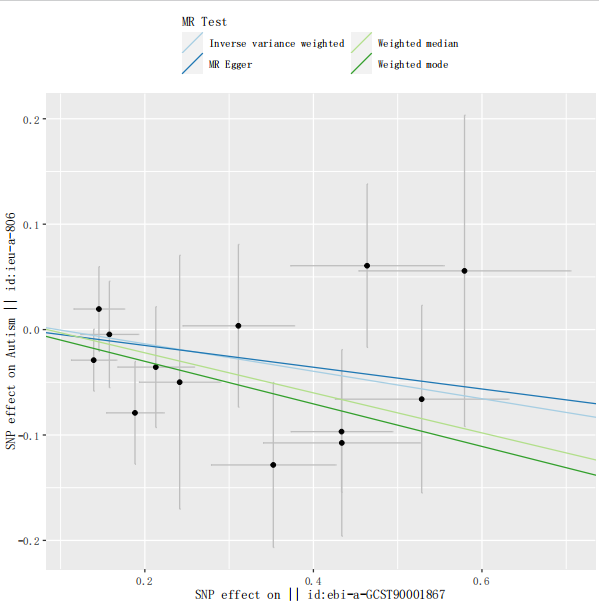

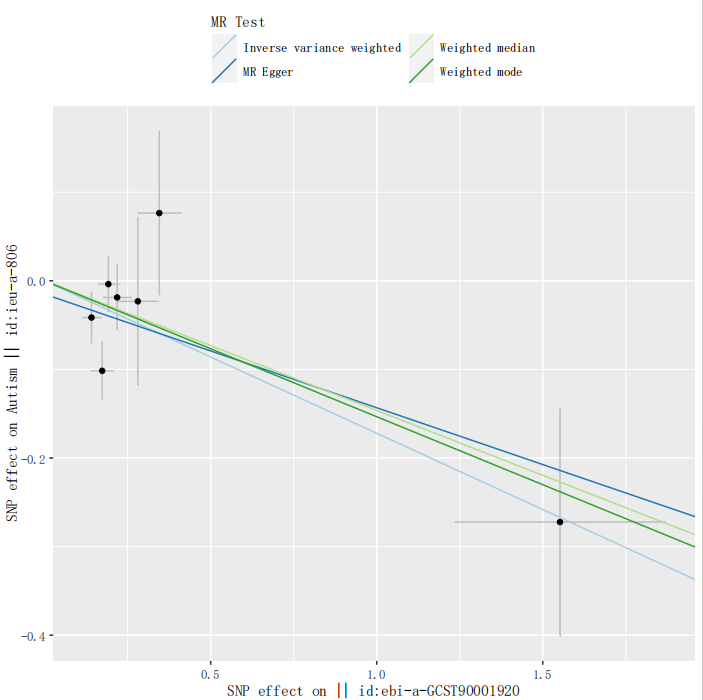

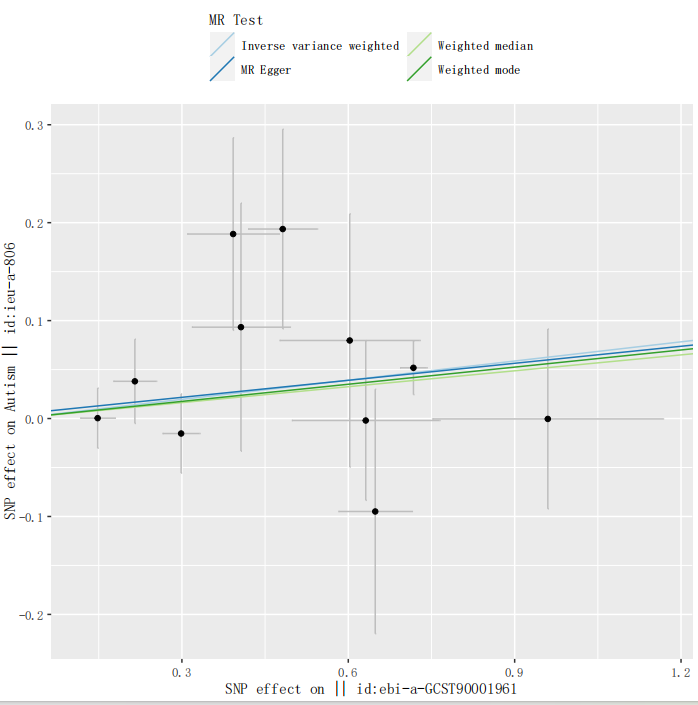


U V W X


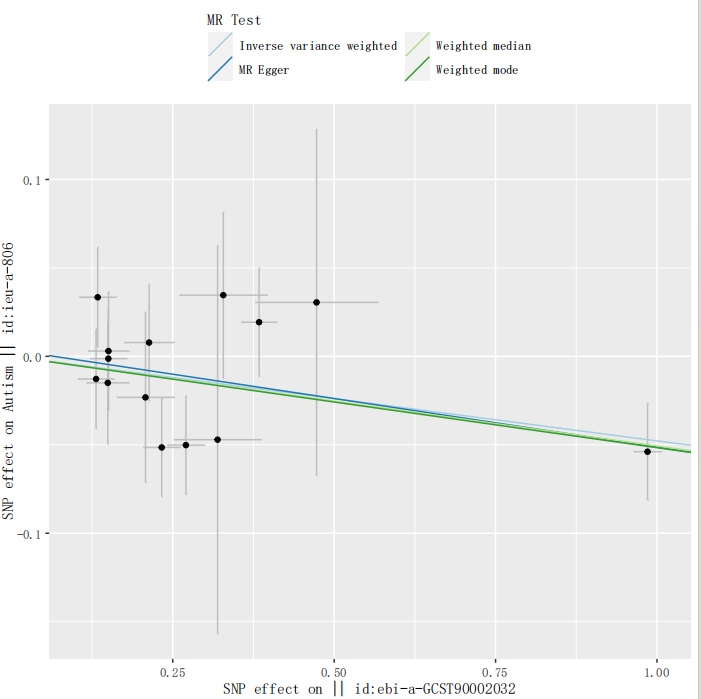

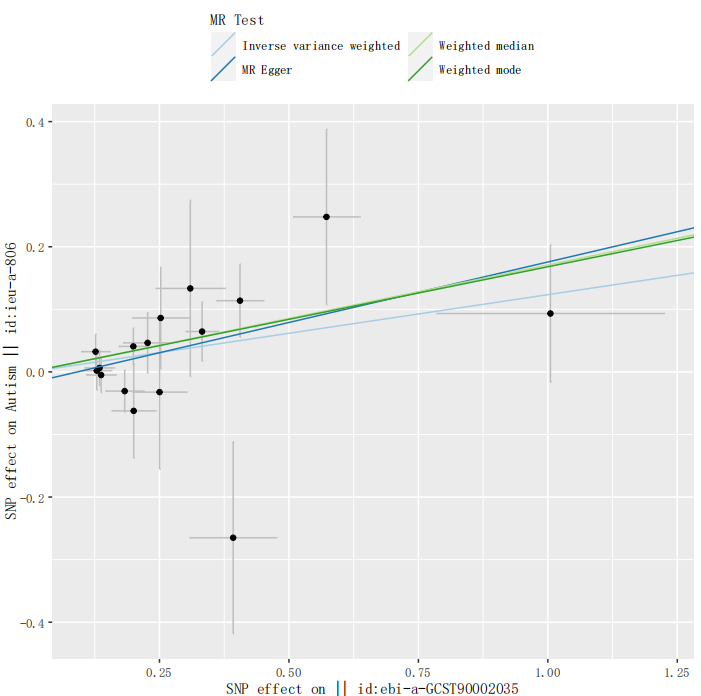

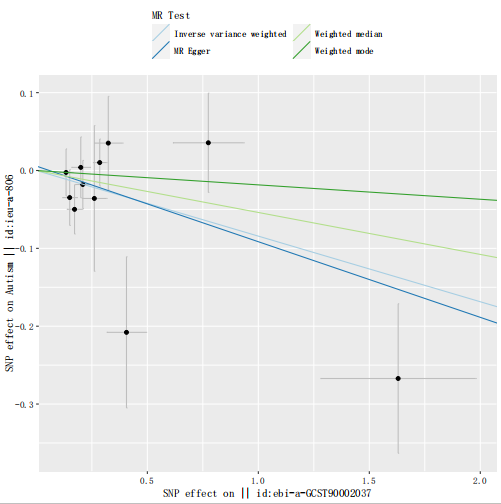

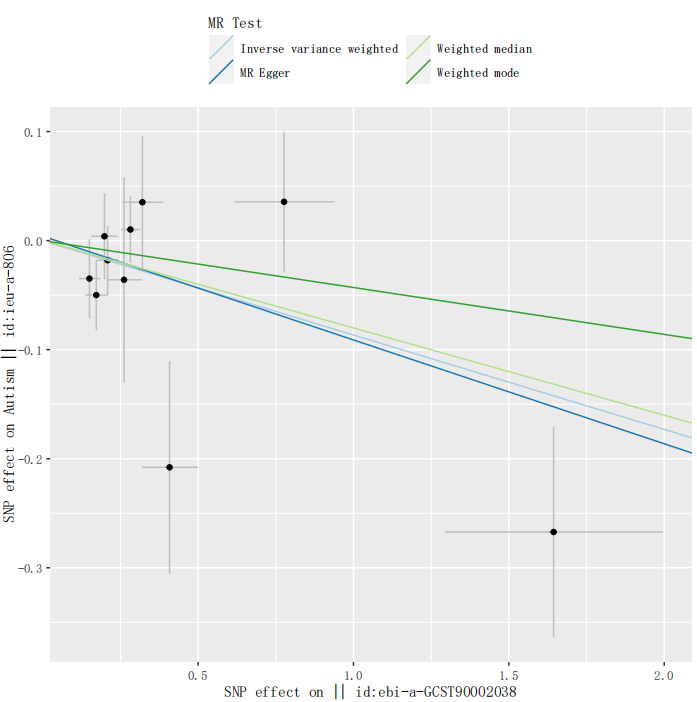


Y Z


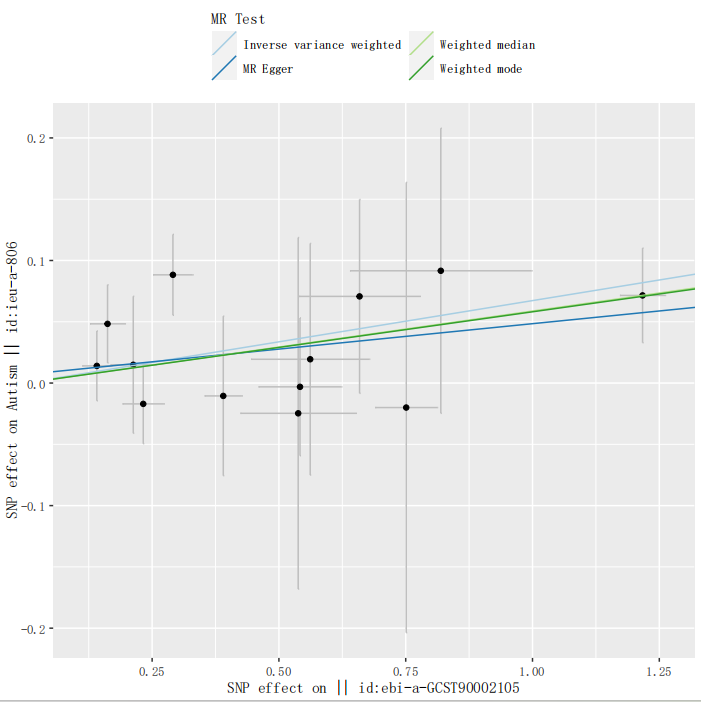

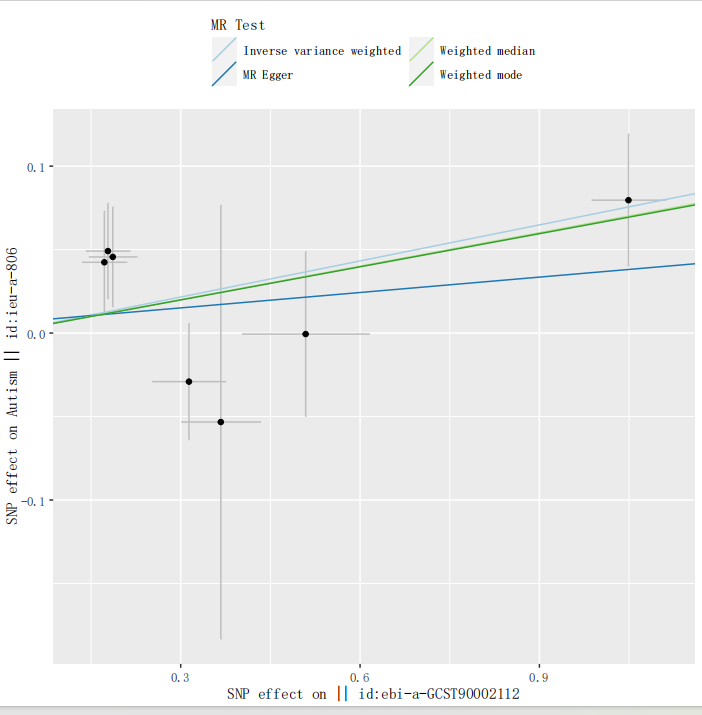


Supplementary Figure 1. Scatter plots for MR analysis of the causal effect of immune cell on ASD,based on the ieu-a-806. (A)Scatter plot between IgD+ CD38br AC and ASD risk; (B) Scatter plot between IgD+ CD24+ %B cell and ASD risk; (C)Scatter plot between CD20- CD38- AC and ASD risk; (D)Scatter plot between CD62L- HLA DR++ monocyte %monocyte and ASD risk;(E)Scatter plot between CD39+ secreting Treg AC and ASD risk; (F)Scatter plot between EM CD4+ %CD4+ and ASD risk; (G)Scatter plot between EM CD8br AC and ASD risk;(H)Scatter plot between CD4+ %leukocyte and ASD risk;(I)Scatter plot between CD3- lymphocyte %leukocyte and ASD risk;(J)Scatter plot between Granulocyte %leukocyte and ASD risk;(K)Scatter plot between CD28- CD8dim %CD8dim and ASD risk;(L)Scatter plot between CD19 on CD20- CD38- and ASD risk;(M)Scatter plot between CD19 on IgD- CD24- and ASD risk;(N)Scatter plot between CD20 on CD24+ CD27+ and ASD risk;(O)Scatter plot between CD20 on IgD+ CD38- and ASD risk;(P)Scatter plot between IgD on IgD+ and ASD risk;(Q)Scatter plot between CD62L on CD62L+ DC and ASD risk;(R)Scatter plot between CD3 on CD4+ and ASD risk;(S)Scatter plot between CD45 on HLA DR+ CD4+ and ASD risk;(T)Scatter plot between CD25 on CD39+ CD4+ and ASD risk;(U)Scatter plot between CD39 on CD39+ CD4+ and ASD risk;(V)Scatter plot between CD80 on myeloid DC and ASD risk;(W)Scatter plot between CD80 on plasmacytoid DC and ASD risk;(X)Scatter plot between CD80 on CD62L+ plasmacytoid DC and ASD risk;(Y)Scatter plot between HLA DR on plasmacytoid DC and ASD risk;(Z)Scatter plot between HLA DR on CD33- HLA DR+ and ASD risk.
