# Supplementary material for: The relationship of immune cells with autism spectrum disorder: a bidirectional Mendelian randomization study
Source: BMC Psychiatry. 2024 Jun 27;24:477. doi: 10.1186/s12888-024-05927-5 (PMC11212275; doi:10.1186/s12888-024-05927-5)
Supplement: Supplementary file 4 — Supplementary Material 4. [file 12888_2024_5927_MOESM4_ESM.docx]

**Supplementary Figures**

A B C D


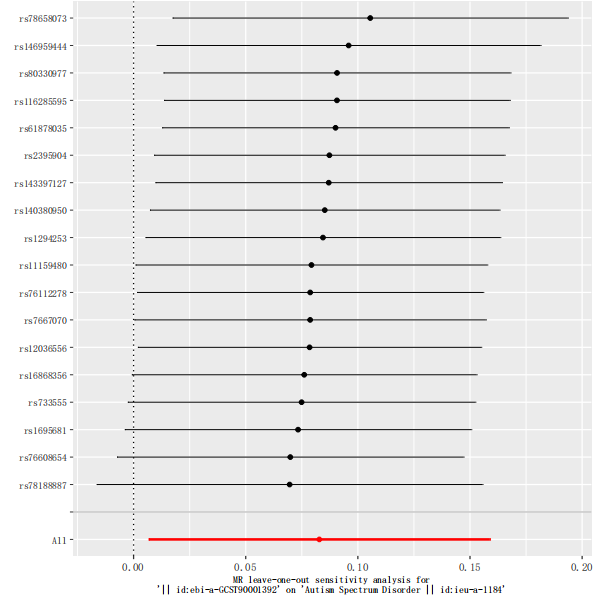

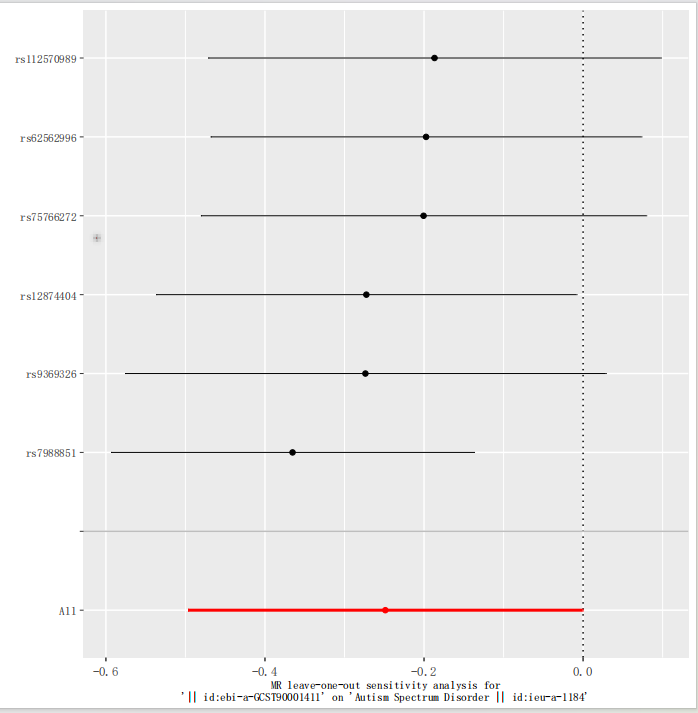

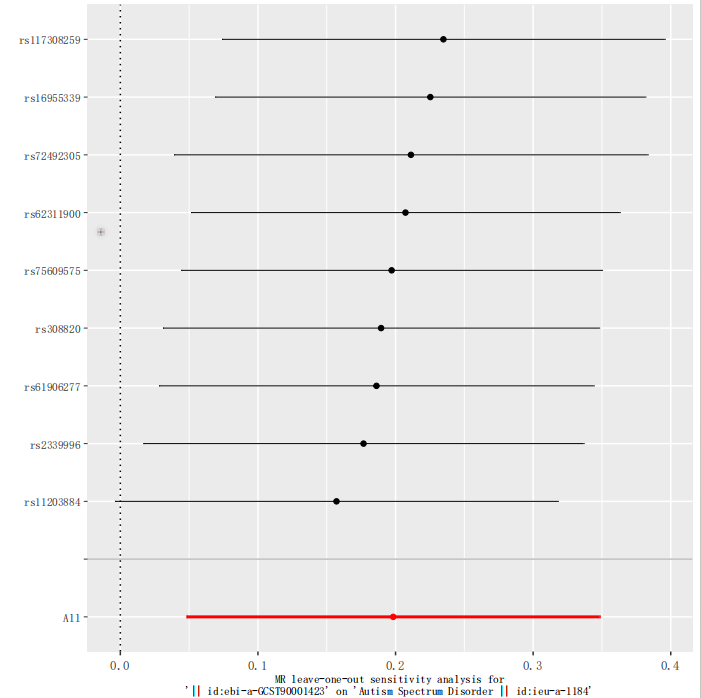

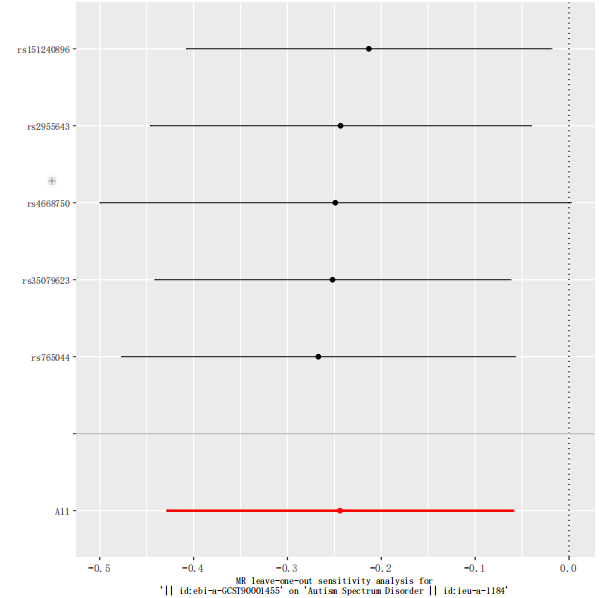


E F G H


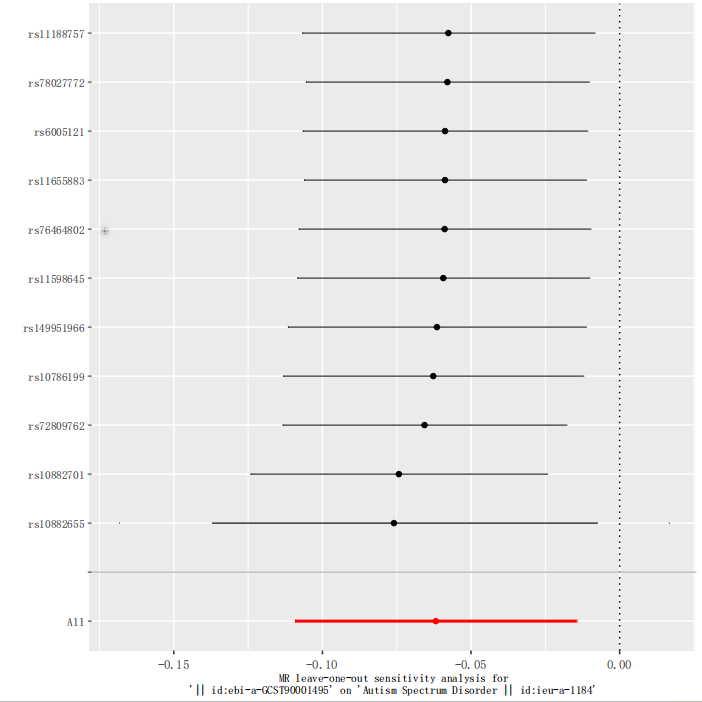

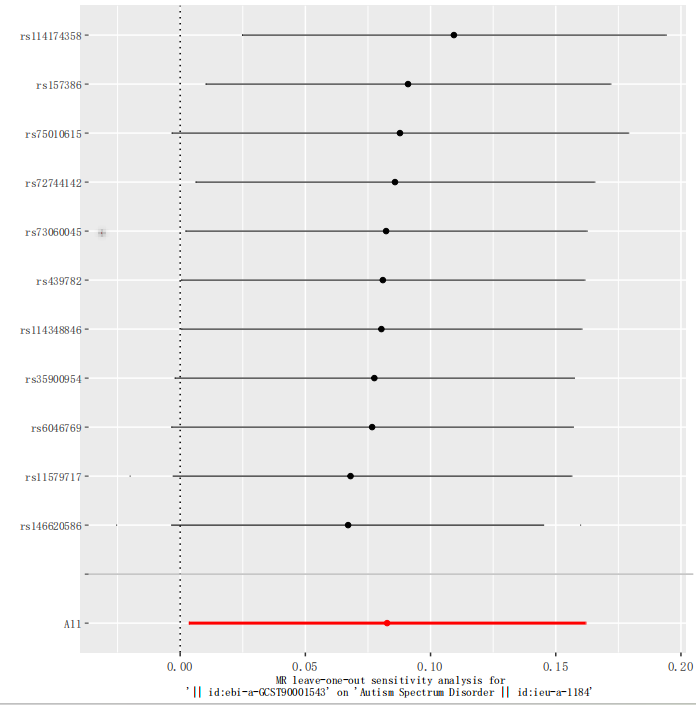

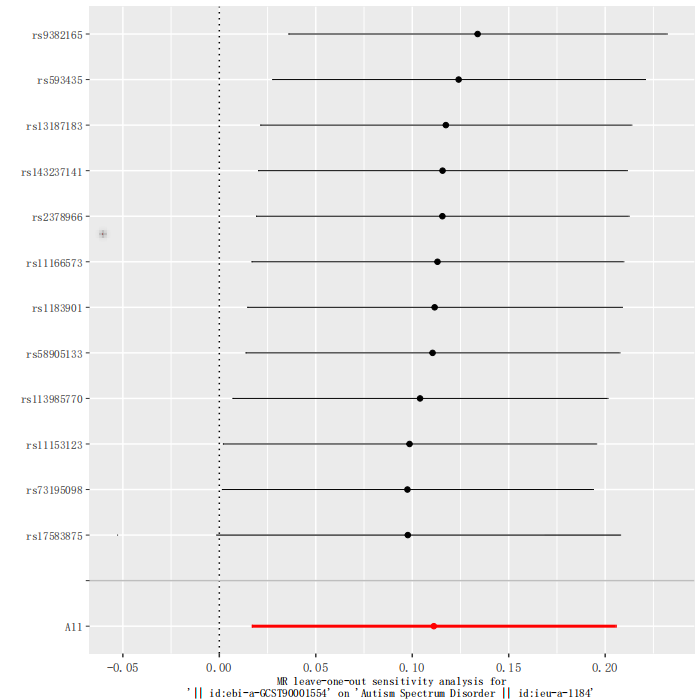

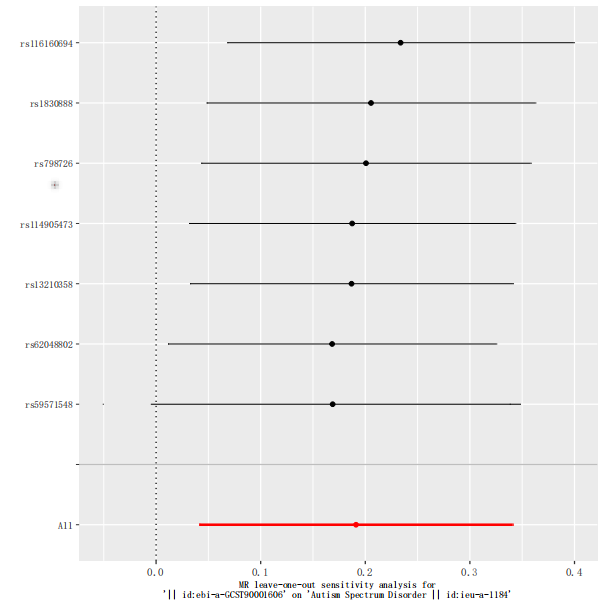


I J K L


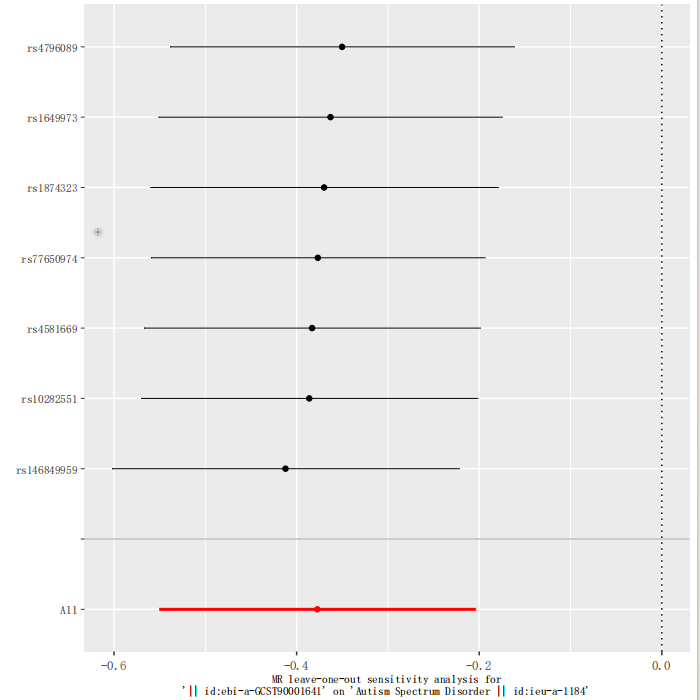

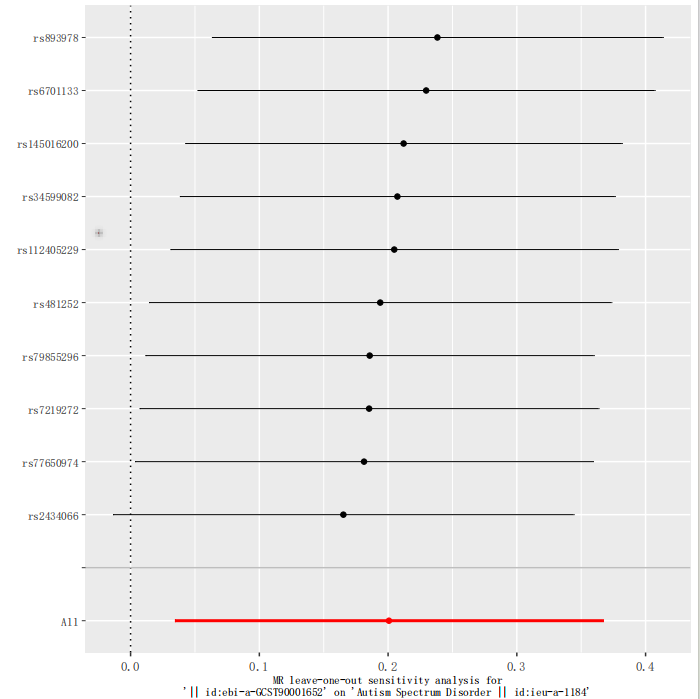

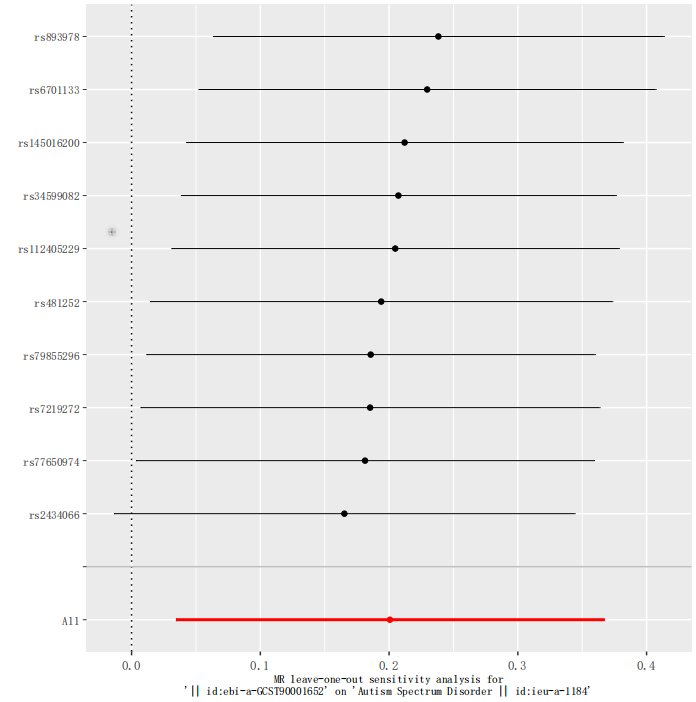

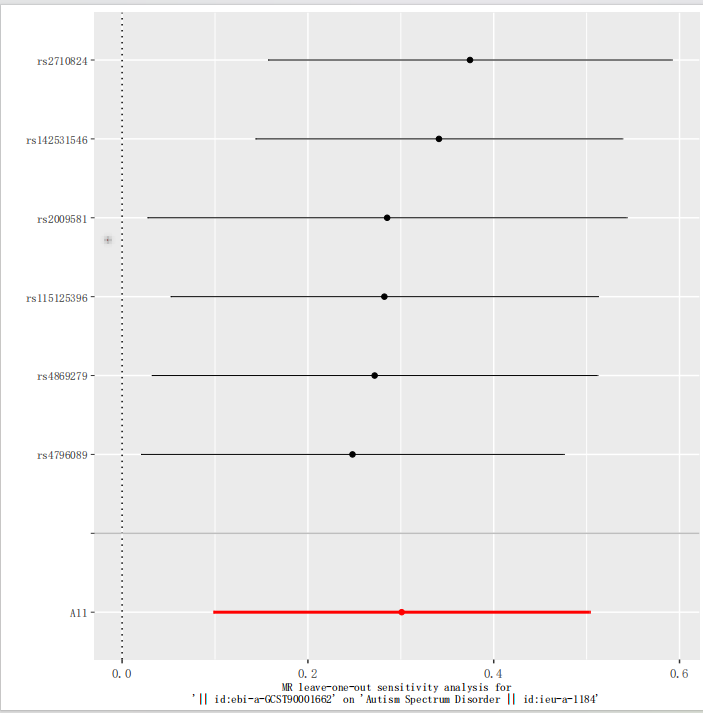


M N O P


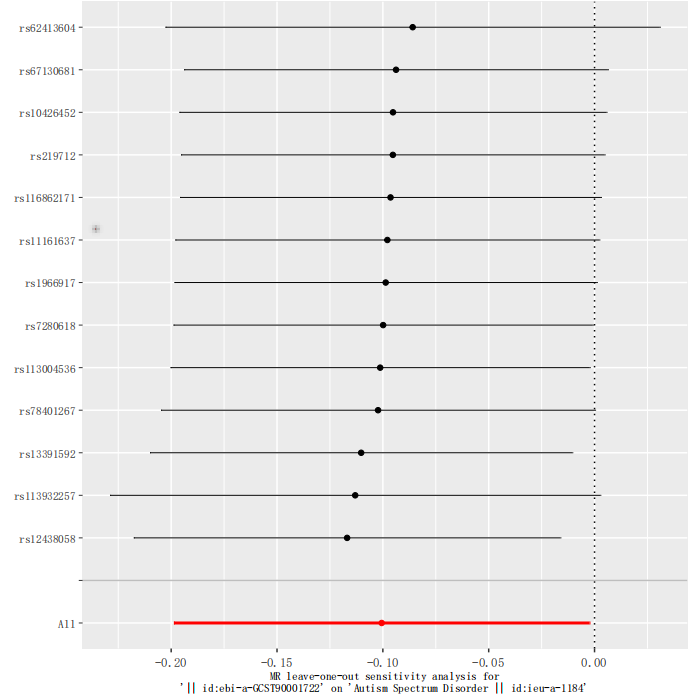

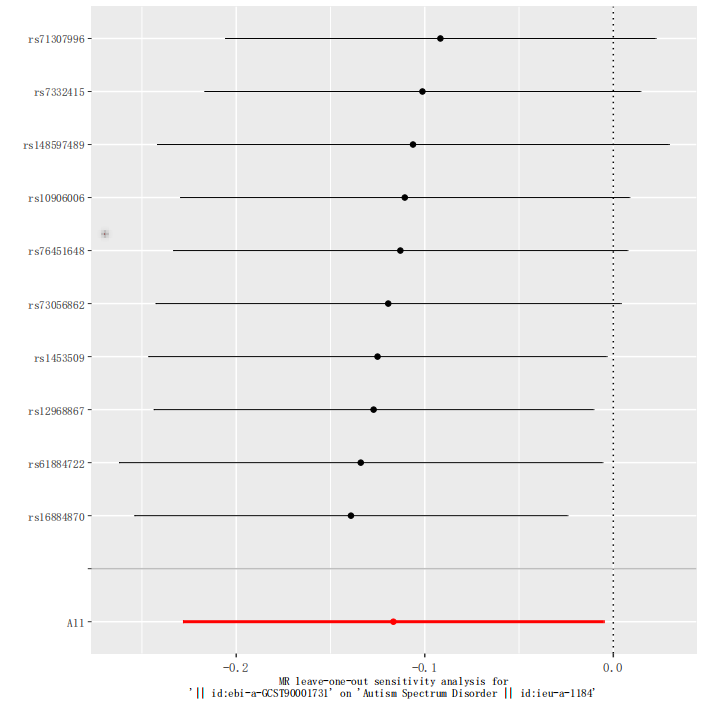

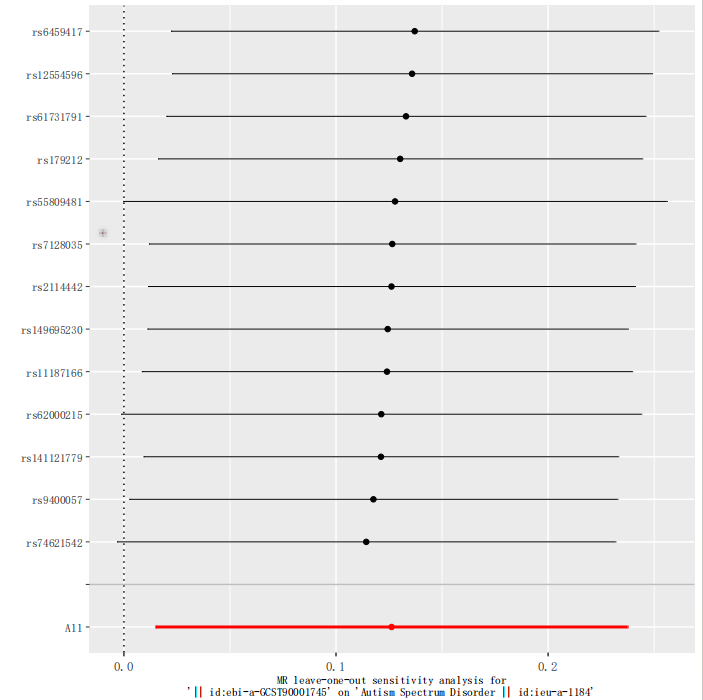

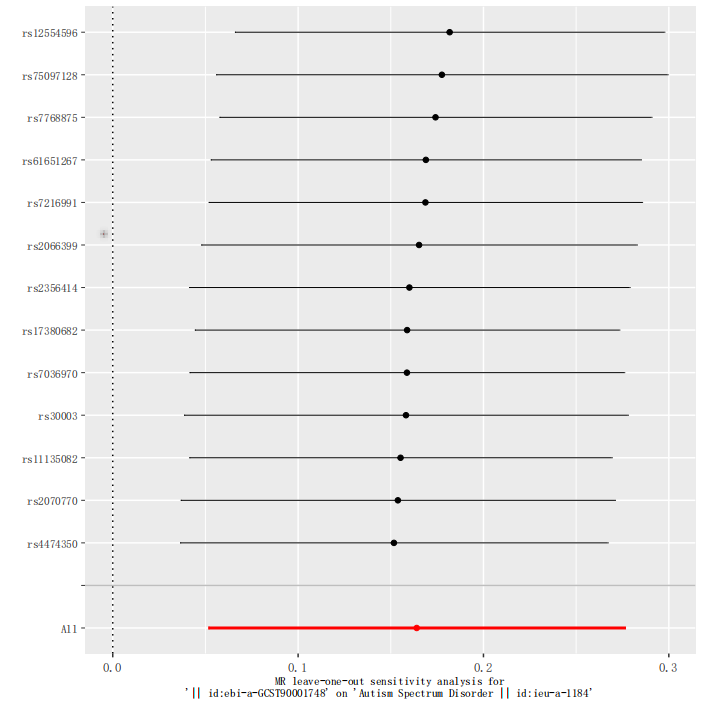


Q R S T


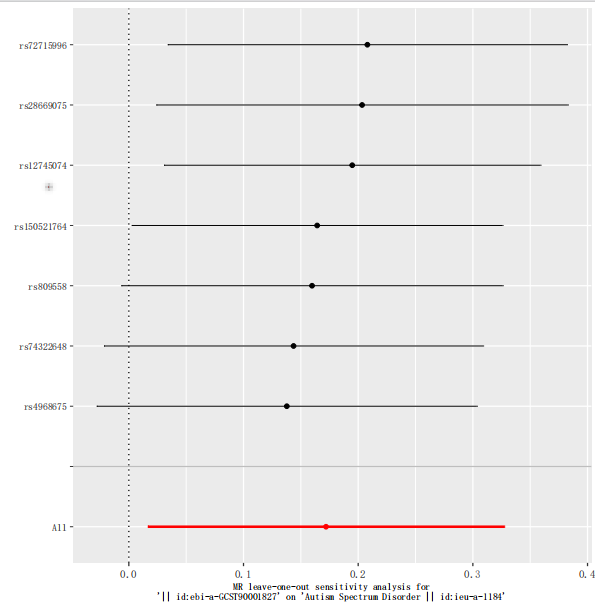

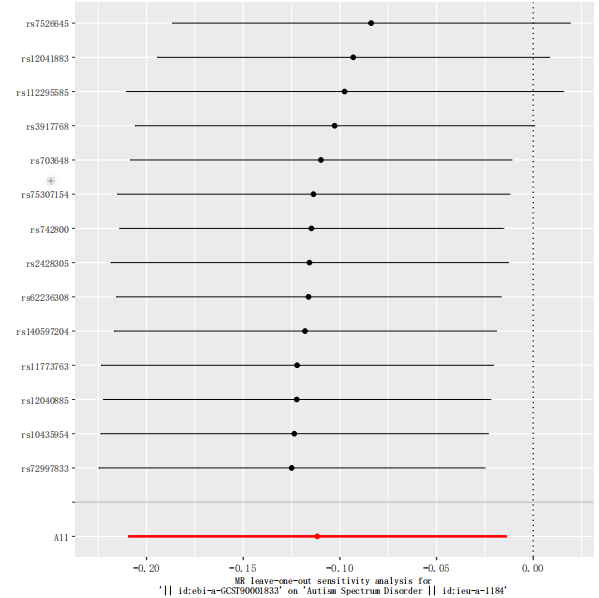

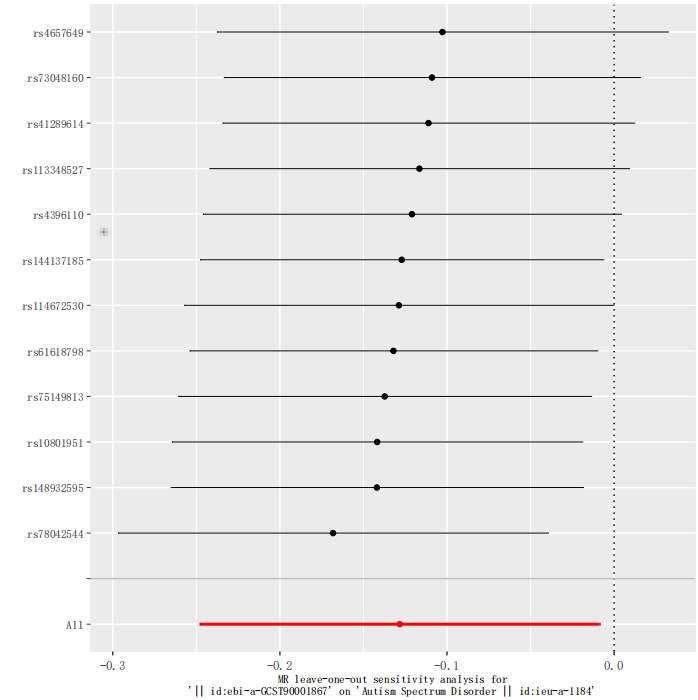

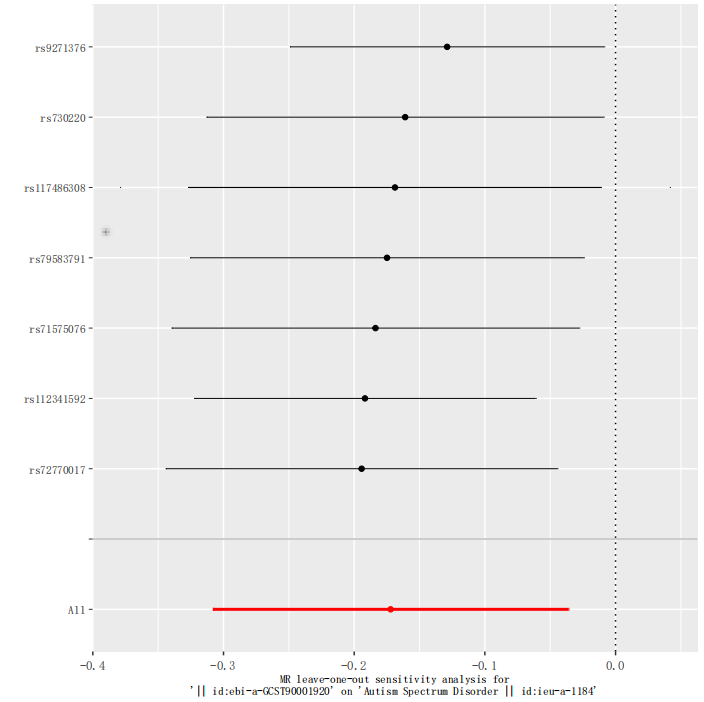


U V W X


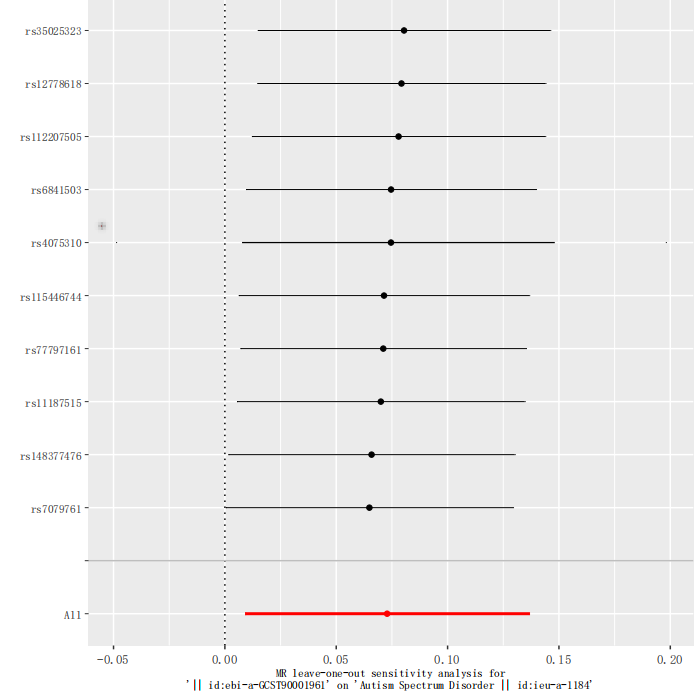

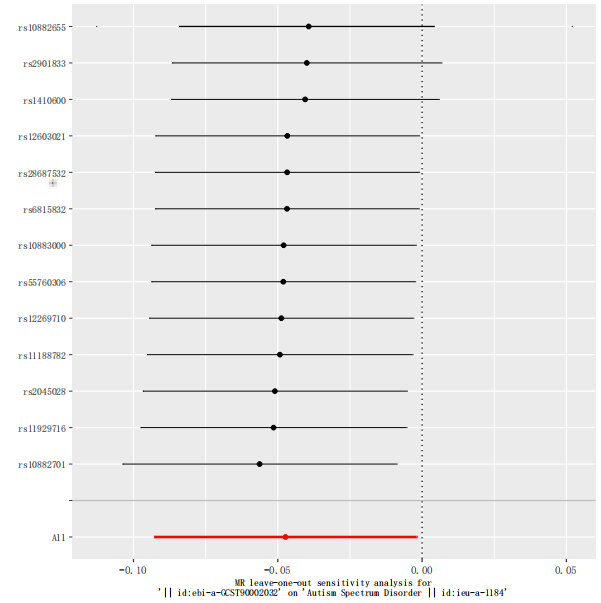

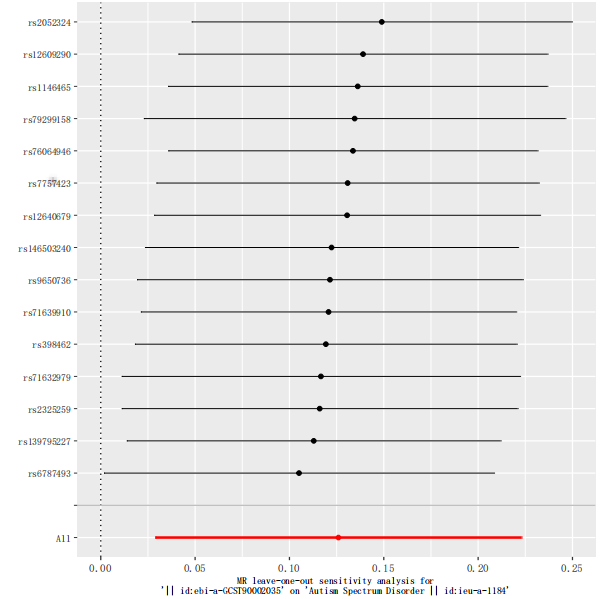

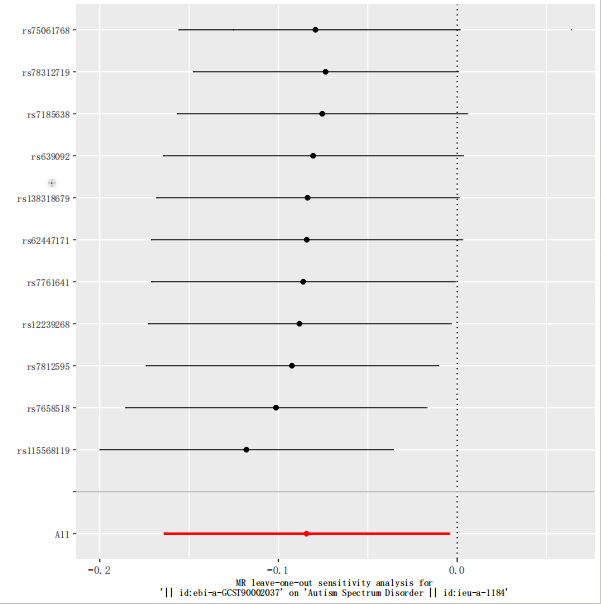


Y Z


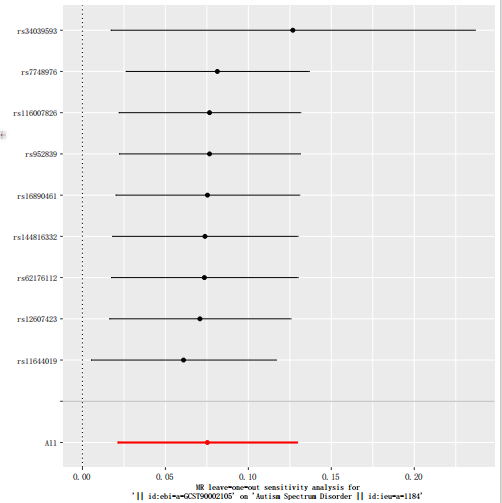

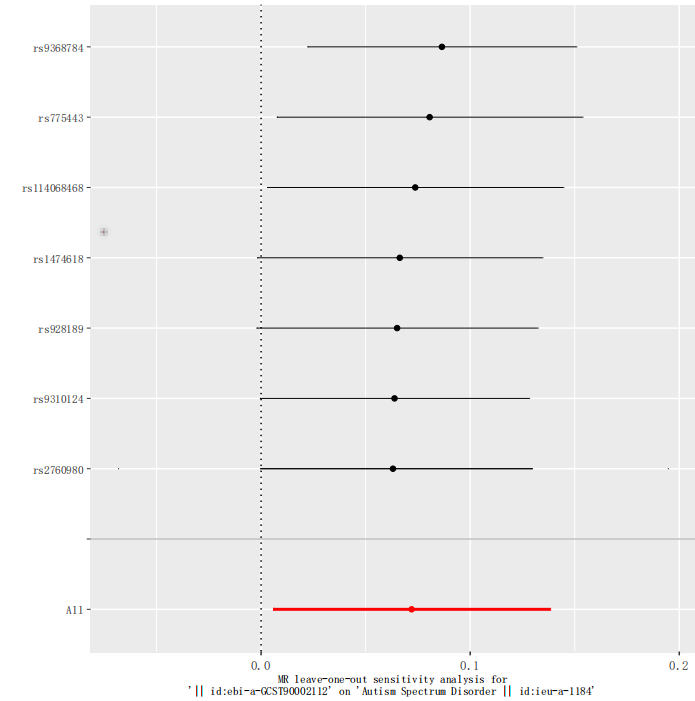


Supplementary Figure4.Leave-one-out analysis for ASD, based on ieu-a-1184.(A)IgD+ CD38br AC;(B)IgD+ CD24+ %B cell;(C)CD20- CD38- AC;(D)CD62L- HLA DR++ monocyte %monocyte;(E)CD39+ secreting Treg AC;(F)EM CD4+ %CD4+;(G)EM CD8br AC;(H)CD4+ %leukocyte;(I)CD3- lymphocyte %leukocyte;(J)Granulocyte %leukocyte;(K)CD28- CD8dim %CD8dim;(L)CD19 on CD20- CD38- ;(M)CD19 on IgD- CD24-;(N)CD20 on CD24+ CD27+;(O)CD20 on IgD+ CD38-;(P)IgD on IgD+;(Q)CD62L on CD62L+ DC;(R)CD3 on CD4+;(S)CD45 on HLA DR+ CD4+;(T)CD25 on CD39+ CD4+ ;(U)CD39 on CD39+ CD4+;(V)CD80 on myeloid DC;(W)CD80 on plasmacytoid DC;(X)CD80 on CD62L+ plasmacytoid DC;(Y)HLA DR on plasmacytoid DC;(Z)HLA DR on CD33- HLA DR+.
